# The small molecule inhibitor BX-795 uncouples IL-2 production from inhibition of Th2 inflammation and induces CD4^+^ T cells resembling iTreg

**DOI:** 10.3389/fimmu.2023.1094694

**Published:** 2023-04-06

**Authors:** Peter A. Tauber, Bernhard Kratzer, Philipp Schatzlmaier, Ursula Smole, Cordula Köhler, Lisa Rausch, Jan Kranich, Doris Trapin, Alina Neunkirchner, Maja Zabel, Sabrina Jutz, Peter Steinberger, Gabriele Gadermaier, Thomas Brocker, Hannes Stockinger, Sophia Derdak, Winfried F. Pickl

**Affiliations:** ^1^ Institute of Immunology, Center for Pathophysiology, Infectiology and Immunology, Medical University of Vienna, Vienna, Austria; ^2^ Institute of Hygiene and Applied Immunology, Center for Pathophysiology, Infectiology and Immunology, Medical University of Vienna, Vienna, Austria; ^3^ Institute for Immunology, Biomedical Center (BMC), Faculty of Medicine, Ludwig Maximilian University (LMU) Munich, Munich, Germany; ^4^ Department of Biosciences, University of Salzburg, Salzburg, Austria; ^5^ Core Facilities, Medical University of Vienna, Vienna, Austria; ^6^ Karl Landsteiner University of Healthcare, Krems, Austria

**Keywords:** Immunomodulation, IL-2, regulatory T cells, small molecule inhibitor, allergy, Th2 cells

## Abstract

**Background:**

Treg cells have been shown to be an important part of immune-homeostasis and IL-2 which is produced upon T cell receptor (TCR)-dependent activation of T lymphocytes has been demonstrated to critically participate in Treg development.

**Objective:**

To evaluate small molecule inhibitors (SMI) for the identification of novel IL-2/Treg enhancing compounds.

**Materials and methods:**

We used TCR-dependent and allergen-specific cytokine secretion of human and mouse T cells, next generation messenger ribonucleic acid sequencing (RNA-Seq) and two different models of allergic airway inflammation to examine lead SMI-compounds.

**Results:**

We show here that the reported 3-phosphoinositide dependent kinase-1 (PDK1) SMI BX-795 increased IL-2 in culture supernatants of Jurkat E6-1 T cells, human peripheral blood mononuclear cells (hPBMC) and allergen-specific mouse T cells upon TCR-dependent and allergen-specific stimulation while concomitantly inhibiting Th2 cytokine secretion. RNA-Seq revealed that the presence of BX-795 during allergen-specific activation of T cells induces a *bona fide* Treg cell type highly similar to iTreg but lacking Foxp3 expression. When applied in mugwort pollen and house dust mite extract-based models of airway inflammation, BX-795 significantly inhibited Th2 inflammation including expression of Th2 signature transcription factors and cytokines and influx into the lungs of type 2-associated inflammatory cells such as eosinophils.

**Conclusions:**

BX-795 potently uncouples IL-2 production from Th2 inflammation and induces Th-IL-2 cells, which highly resemble induced (i)Tregs. Thus, BX-795 may be a useful new compound for the treatment of allergic diseases.

## Introduction

Interleukin-2 is an important T cell growth factor (1) that critically contributes to immune homeostasis by promoting development and expansion of regulatory T cells (2–7). In fact, several strategies used IL-2 to expand regulatory T cells to treat autoinflammatory and allergic diseases. Examples include IL-2/anti-IL-2 antibody complexes that extend the half-life of IL-2 and contribute to its improved targeting (8, 9), and IL-2 muteins with improved IL-2R specificity, both being the subject of preclinical (10) or clinical studies (11). IL-2 exerts its Treg-promoting activity *via* binding to the high-affinity IL-2 receptor (CD25) (3, 4, 7, 12). Spontaneous mutations in the human IL-2RA (CD25) gene (13) or genetic ablation of the mouse *il2ra* locus result in Treg deficiency (7, 12), uncontrolled T cell expansion (14), and overt autoimmunity (13, 15), a clinical picture similar to the immunodysregulation polyendocrinopathy enteropathy X-linked (IPEX) syndrome in which Foxp3 (16), the signature transcription factor of Treg cells, is dysfunctional (17, 18).

Given the importance of IL-2 (19, 20), we were interested in identifying targeted drugs that would interfere with aspects of the TCR-signaling pathway and increase secreted IL-2 levels from T cells. We hypothesized that such drugs would concomitantly limit cellular activation/proliferation of T cells. Previous reports have shown that this constellation best promotes conversion of naïve T cells to Treg cells (21–24). Accordingly, we assumed here that partial inhibition of proliferation in the presence of elevated/induced IL-2 levels could promote Treg formation and thus should be an appropriate guide for our mining strategy to identify interesting lead substances (19, 25).

Therefore, in Jurkat E6-1 cells (26, 27) we initially evaluated TCR cell signaling pathway SMI such as the Src-kinase inhibitor Saracatinib (28), the Erk-2 inhibitor Vx-11e (29), the calcineurin inhibitors Tacrolimus (30) and Cyclosporin A (31), the c-Jun-N-terminal-kinase inhibitor SP600125 (32), the mTORC1/2 inhibitor AZD8055 (33) and the PDK-1/TBK-1 inhibitor BX-795 (34) for their potential IL-2-inducing activity. Our screen revealed BX-795 as unique in its capacity to enhance IL-2 production in Jurkat E6-1 T cells upon stimulation. We went on to extensively explore the immunomodulatory properties of BX-795 in human and murine T cells using human peripheral blood mononuclear cells (PBMC) activated by CD3 antibody or superantigen from *Staphylococcal* enterotoxin A and allergen-specific T cells from humanized allergy mice (35). *In vivo* studies in these humanized allergy mice and in a house dust mite (HDM)-based mouse model of allergen-specific airway inflammation confirmed the *in vivo* allergy-mitigating effect of BX-795 and together with gene expression analyses established the definition of a putatively new Th cell phenotype.

## Materials and methods

### Stimulation of Jurkat E6-1 T cells for cytokine production

Jurkat E6-1 cells (ATCC, Manassas, VA, USA) were incubated on CD3 coated plates in the presence or absence of the indicated SMI for 24 hours. After centrifugation (500 g, 5 minutes) of plates, 150 µL supernatant was harvested and subjected to cytokine analyses as shown previously (36).

### RNA-Seq and data analysis

Preparation of sequencing libraries, QC-check and sequencing from naïve or differentially activated allergen-specific T cells were performed as described (37), obtaining 32 million reads per sample, which were aligned to the mouse reference genome version GRCm38 (38) with Gencode mV23 annotations (39) using STAR aligner (40) version 2.6.1a in 2-pass mode. Reads per gene were counted by STAR, differential gene expression was calculated using DESeq2 (41) version 1.22.2.

### Animal experimental procedures

TCR/DR1 double transgenic mice expressing a human major mugwort pollen allergen Art v 1_25-36_ peptide-specific TCR in the context of HLA-DR1 (35) were cohoused with C57BL/6J wild type mice at the Medical University of Vienna, Vienna, Austria. TCR/DR1 mice were used as source for allergen-specific T cells and antigen presenting cells and for *in vivo* challenge experiments with mugwort pollen extract (MPE). Experimental procedures were approved by the Institutional Review Board and the Federal Ministry of Science, Research and Economy (GZ : BMBWF-66.009/0288-V/3b/2018). Mice were free of mouse pathogenic viruses, bacteria and parasites (42).

### Statistical analyses

Normally distributed data were compared using parametric tests (Student’s t-test or one-way ANOVA) followed by correction of alpha (Dunnett, Tukey or Holms-Sidak) using GraphPad 9.0.1 (GraphPad Software Inc., La Jolla, CA). Otherwise, the Mann-Whitney U-test or the Kruskal-Wallis test was performed, followed by Dunn’s multiple comparison testing. ns, not significant; *, p < 0.05; **, p < 0.01; ***, p <0.001.

Further materials and methods and experimental details can be found in the Supplementary Information.

## Results

### The small molecule inhibitor BX-795 increases transcription and secretion of IL-2 in TCR/CD3-stimulated Jurkat E6-1 T cells

**Figure 1:**
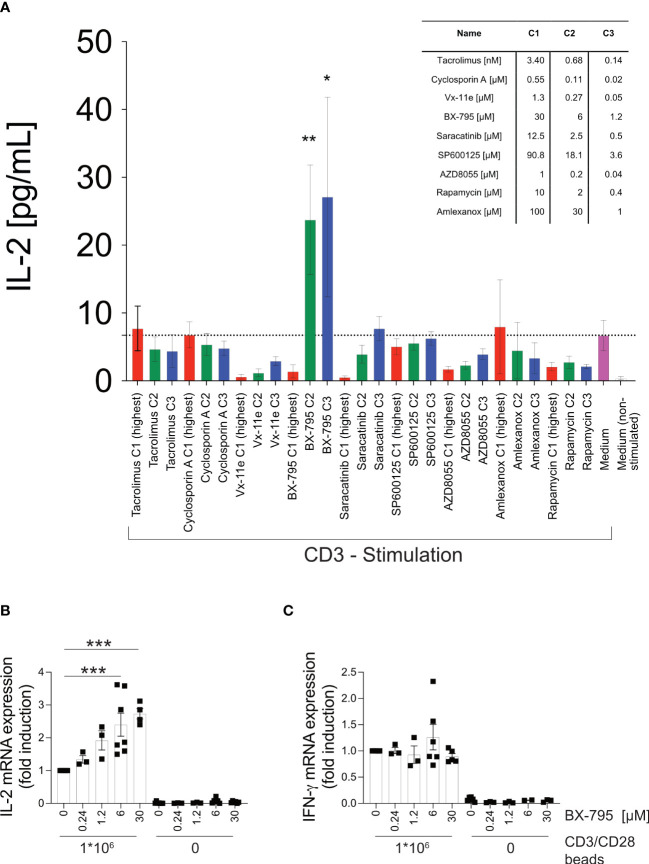
T cell activation studies identify BX-795 as a novel, IL-2 enhancing SMI which acts independently of accessory cells. Shown are **(A)**, IL-2 levels in supernatants of Jurkat E6-1 cells (1x10^5^ cells/well) incubated in 96-well flat bottom plates in the presence (colored bars) or absence (white bar) of plate-bound CD3 mAb OKT3 in the presence (red, green, blue) or absence (magenta and white bar) of the indicated SMI applied at the indicated final concentrations C1 – C3 (see inset table, C1 red, C2 green, C3 blue). All conditions contained DMSO at a final concentration of 0.25% (v/v). Shown are relative **(B)**, IL-2 and **(C)**, IFN-γ mRNA expression levels as assessed by RT-qPCR of Jurkat E6-1 cells (1x10^6^ cells/well in a 48 well plate, 400 µL final volume) incubated in the presence or absence of CD3/CD28 beads (1x10^6^ beads/well) and the indicated concentrations of BX-795 for 6 hours in 400 µL final volume. Specific mRNA levels were first normalized to the housekeeping gene β-2 microglobulin (β2M) and then normalized to the mean of the CD3/CD28 beads stimulated DMSO condition for each respective experiment. All conditions contained DMSO at a final concentration of 0.25% (v/v). Data show in A, mean values ± SEM of four independently performed experiments (except three for rapamycin, and two for amlexanox) tested in triplicates and in B, the summary (means ± SEM) of seven (except 3 for 0.24 and 1.2 µM; 4 for CD3/CD28 at 30 µM; and 6 for unstimulated 6 and 30 µM) independently performed experiments is shown. In **(C)**, the summary (means ± SEM) of six (except 5 for 30 µM; 3 for 0.24 and 1.2 µM; and 2 for unstimulated at 6 µM) are shown. One-way ANOVA with Dunnett’s correction for multiple comparisons **(A)**, comparing all conditions against CD3 stimulated cells in solvent (mean of CD3 stimulated control indicated by dotted line) and **(B, C)**, comparing the respective stimulated and BX-795 treated condition to the stimulated condition which was treated with solvent only. Statistically significant changes are indicated with *, p < 0.05; **, p < 0.01; ***, p < 0.001.

Herein, we tested a collection of SMI (**Table S1**) for their IL-2-inducing effects in the context of TCR-dependent T cell activation that simultaneously had some effect on T cell growth (25). We found that the putative PDK-1/IKKε/TBK-1 multikinase inhibitor BX-795 significantly increased secreted IL-2 levels at 1.2 and 6 µM (27.1 ± 14.7 and 23.8 ± 8.1 versus 6.71 ± 2.23 pg/mL, mean ± SEM) (**Figure 1A**), decreased soluble IL-2R alpha (CD25) levels (**Figure S1A**) but maintained cellular viability (**Figure S1B**) of the human leukemic T cell line Jurkat E6-1 (26) upon TCR/CD3 ligation. In accordance with the increased IL-2 protein levels observed after 24 hours of stimulation, Jurkat E6-1 cells stimulated with CD3/CD28 beads for 6 hours, highly significantly increased IL-2 (**Figure 1B**) but not IFN-γ (**Figure 1C**) mRNA levels with a clear trend towards increased IL-2 mRNA already at 0.24 µM of BX-795. In contrast, at 30 µM BX-795 showed toxic effects after 24 hours (**Figure S1B**), while the same concentration clearly induced elevated IL-2 mRNA levels after 6 hours (**Figure 1B**). Neither treatment with the TBK-1/IKKε inhibitor amlexanox (**Figure 1A**) (43) nor with six other SMI revealed a similar IL-2 hypersecretion phenotype, while several SMI dose-dependently reduced TCR/CD3-induced IL-2 production (*e.g.*, Vx-11e, Saracatinib, and AZD8055) (**Figure 1A**). These results suggested that both increased synthesis and release but also reduced consumption of IL-2 may contribute to increased IL-2 levels in TCR/CD3-stimulated Jurkat E6-1 cells in the presence of BX-795. Next, we tested whether the BX-795-induced IL-2 secretion was the result of increased TCR proximal phosphorylation and/or Ca^2+^-fluxing (**Figure S2**). Interestingly, BX-795 reduced TCR-induced intracellular Ca^2+^-fluxing in Jurkat E6-1 and primary human T cells with different sensitivity (**Figures S2A, B**), which may be related to early attenuation of tyrosine-phosphorylation by BX-795 (as determined with the anti-pan tyrosine-phosphorylation antibody 4G10) (**Figure S2**). However, BX-795 sustained overall (**Figure S2C**), Lck-specific (**Figure S2D**) and CD3 ζ-chain-specific (**Figure S2E**) tyrosine-phosphorylation at later time points, while it left Erk tyrosine-phosphorylation unaffected (**Figure S2F**) when compared to the solvent (DMSO). These changes at early time points were also quantified using densitometric analyses of the indicated lanes of the Western blots (**Figure S2G**).

### BX-795 co-stimulates IL-2 but mitigates Th2 cytokine secretion in PB T cells of healthy donors

**Figure 2:**
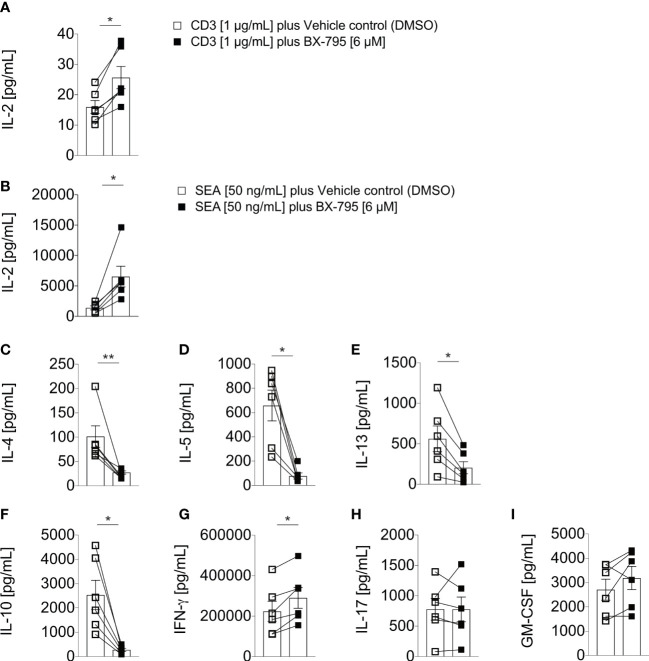
BX-795 stimulates IL-2 but inhibits Th2 effector cytokine secretion in primary human peripheral blood T cells. Shown are **(A, B)**, IL-2; **(C)**, IL-4; **(D)**, IL-5; **(E)**, IL-13; **(F)**, IL-10; **(G)**, IFN-γ; **(H)**, IL-17; and **(I)**, GM-CSF; levels in supernatants of human PBMC (1x10^5^/well) which were stimulated in 96-well round bottom tissue culture plates with soluble 1 µg/mL CD3 mAb in A, or 50 ng/mL SEA in B to I in the presence or absence of 6 µM BX-795 for 72 hours. All cultures contained DMSO at a final concentration of 0.25% (v/v). Shown are A to I, the summaries (mean values ± SEM) of secreted cytokine levels obtained with PBMC of six unrelated healthy individuals analyzed in two independently performed experiments. The non-stimulated baseline levels for the indicated cytokines for medium plus DMSO and medium plus BX-795 solubilized in DMSO were (mean ± SEM): IL-2: 10.1 ± 3.1 and 8.8 ± 3.7; IL-4: 23.5 ± 2.6 and 26.7 ± 3.3; IL-5: 1.6 ± 1.0 and 0.8 ± 0.1; IL-13: 3.9 ± 1.8 and 2.3 ± 0.5; IL-10, 6.9 ± 1.3 and 8.8 ± 1.8; IFN-γ: 233.7 ± 63.9 and 111.3 ± 43.0; IL-17A: 1.2 ± 0.3 and 1.2 ± 0.2 and GM-CSF: 16.0 ± 3.9 and 7.9 ± 3.2; respectively. Paired two-tailed Mann-Whitney U-test. Only statistically significant changes are indicated with *, p < 0.05; **, p < 0.01.

Next, we examined human PBMC co-incubated with CD3 mAb (OKT3) or the *Staphylococcus aureus* superantigen A (SEA) in the presence or absence of BX-795. We observed that BX-795 significantly increased IL-2 levels in OKT3 (25.7 ± 9.0 versus 16.0 ± 5.2 pg/mL, mean ± SEM; 1.6-fold) and SEA (6515.0 ± 4153 versus 1368.0 ± 811.1 pg/mL, mean ± SEM; 4.76-fold) stimulated T cells (**Figures 2A, B**) while it inhibited Th2 cytokine and the regulatory cytokine IL-10 secretion levels (**Figures 2C–F**). Moreover, BX-795 treatment slightly increased IFN-γ but left GM-CSF and IL-17a secretion levels unchanged (**Figures 2G–I**). The contribution of BX-795 to enhanced IL-2 secretion was corroborated with murine allergen-specific transgenic T cells (35). T cells from these mice express a human TCR, which is specific for the human-relevant, immunodominant major mugwort pollen allergen Art v 1_25-36_ peptide in the context of HLA-DR1. Upon stimulation of these T cells with the cognate Art v 1_23-36_ peptide in the presence but not in the absence of BX-795, the levels of IL-2 were likewise significantly increased (**Figure S3A**), while the levels of the Th2 cytokines IL-4, IL-5 and IL-13 (**Figures S3B–D**), and that of the regulatory cytokine IL-10 (**Figure S3E**) were reduced. Similar to human T cells, detectable GM-CSF and IL-17 levels in supernatants remained unchanged in the presence of BX-795 in murine T cells as well (**Figures S3F, G**), while the Th1 cytokine IFN-γ was reduced (**Figure S3H**). **Figure S3I** shows that the changes in cytokine expression levels were TCR-signal- (peptide concentration) and time-dependent.

BX-795 has been previously described as an IKKε inhibitor (44). IKKε was shown to be involved in an inhibitory feedback loop controlling NFATc1 translocation and IL-2 production (45). Therefore, we speculated that BX-795 might act in a similar fashion on NFATc1 translocation as an explanation for increased IL-2 secretion. However, we found no indication for increased NFATc1 translocation preceding the elevated IL-2 levels neither upon BX-795 nor upon treatment with the recently described TBK-1/IKKε inhibitor amlexanox (43) of murine T cells compared to solvent control (**Figure S4**), suggesting that NFATc1 translocation was not crucial for BX-795 mediated IL-2 enhancement.

At higher concentrations, BX-795 gradually impacted on the proliferation of human and murine T cells, which may contribute to the salient features of the induced T cell phenotype (**Figure S5**). Consistent with its impact on primary allergen-specific murine and human polyclonal T cells, BX-795 also significantly inhibited allergen-specific recall responses (**Figure S6**). In fact, upon restimulation of mugwort major pollen allergen Art v 1-specific T cells with the cognate allergen-specific peptide, BX-795 significantly reduced numbers of IL-2^-^IL-13^+^ Th2 cells (**Figure S6B**), residual IL-2^-^IFN-γ^+^ Th1 cells (**Figure S6H**) and cellular proliferation (**Figure S6G**), while IL-2^+^IL-13^-^ T cells were found modestly increased (**Figure S6D**). This suggested that BX-795 is capable of inhibiting Th2 cytokine production even in already differentiated, actively cycling Th2 cells, which argues against the possibility that BX-795 merely inhibits Th2 differentiation in primary non-differentiated cells.

Taken together, antigen (allergen)-dependent T cell activation in the presence of BX-795 favors the differentiation of a putatively novel, IL-2-secreting T helper cell phenotype, here referred to as Th-IL-2 cells, that consistently turns off Th2 cytokine production in human and murine T cells.

### Th-IL-2 cells are phenotypically highly similar to iTreg

**Figure 3:**
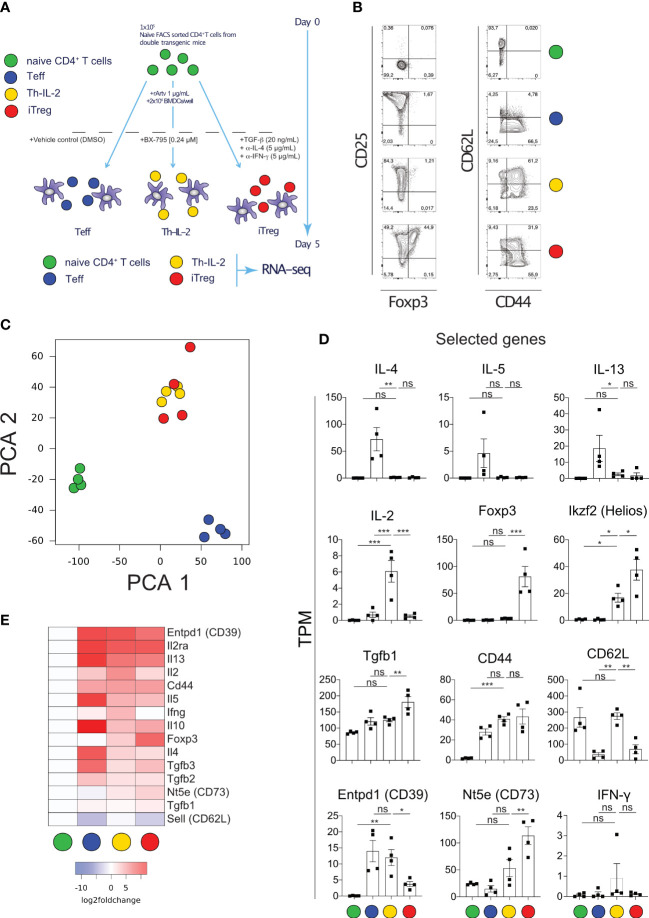
Transcriptomic analyses of naïve T cells activated allergen-specifically in the presence of BX-795 reveals a Th-IL-2 cell phenotype which is distinct from but has close similarities with iTregs. **(A)**, shown is the experimental scheme for allergen-specific T cell activation. For each experimental condition and the numbers of experiments performed a colored dot is assigned. **(B)**, shown are representative FACS plots of freshly sorted naïve (0 h) and differentially activated (120 h) CD3^+^CD4^+^ T cells upon culture at the conditions shown in **(A)**. **(C)**, shown are the results of the principal component analyses (PCA) of the RNA-Seq data derived from viable and naïve (0 h) and differentially activated (120 h) CD3^+^CD4^+^ T cells according to **(A)**. Colors indicate the respective experimental condition, dots the results of the independently performed experiments. **(D)**, shown are bar graphs (mean values ± SEM) summarizing the expression of selected genes for the four experimental conditions as transcripts per million (TPM). Data shown are representative for one out of 4 experiments in **(B)** and show the mean value ± SEM of four independently performed experiments in **(D)**. In **(D)**, statistical analysis was performed with one-way ANOVA with Dunnett’s correction of multiple comparisons. All comparisons performed against the condition Th-IL-2. **(E)**, shown is a heat-map displaying the expression of manually curated T helper cell signature genes. Statistically significant changes are indicated with *, p < 0.05; **, p < 0.01; ***, p < 0.001. ns, non-significant.

To determine and relate the phenotype of BX-795-induced Th-IL-2 cells to well-defined effector and regulatory T cells, RNA-Seq experiments were carried out. For that purpose, naïve allergen-specific murine CD4^+^CD44^-^CD62L^+^ T cells were stimulated with recombinant Art v 1 protein in the presence of bone marrow derived dendritic cells (BMDC) and either solvent alone (T_eff_ cells), BX-795 (Th-IL-2 cells) or exogenous TGF-β1 plus anti-IFN-γ and anti-IL4 mAbs (iTreg) according to the scheme shown in **Figure 3A**. Due to the higher sensitivity of naïve FACS-sorted T cells, these experiments had to be carried out with a lower (0.24 µM) concentration of BX-795. Flow cytometry analyses revealed that induced(i)Tregs upregulated CD25 and Foxp3. However, Th-IL-2 cells, similar to Teff, lacked Foxp3 expression, although they were comparably activated as shown by > 60% of cells neo-expressing CD44 (**Figure 3B**). In addition, Th-IL-2 cells revealed a decreased expression in CD25. Despite the clear difference in Foxp3 expression, principal component analyses of RNA-Seq data revealed that Th-IL-2 cells strongly co-clustered with iTreg cells, but were clearly distinct from T_eff_ and naïve Th cells (**Figure 3C**). Consistent with the cytokine secretion results, mRNA levels of Th-IL-2 cells were high for *Il2* but low for the Th2 cytokine genes *Il4*, *Il5* and *Il13*, which was exactly opposite to Teff cells (**Figure 3D**). Compatible with the flow data and in contrast to iTregs, Th-IL-2 cells were devoid of *Foxp3* message while they expressed appreciable mRNA-levels of *Ikzf2* (Helios), *Nt5e* (CD73) and *Entpd1* (CD39), all well-established signature genes of Treg cells (16, 46–48) (**Figure 3E**). RNA-Seq data for *Foxp3*, *Ikzf2* and *Il4* genes were confirmed by qPCR (**Figure S7**). The observed increase in IFN-γ mRNA expression levels for Th-IL-2 cells was unexpected (**Figure 3E**), however, the increase was not significant and attributable to one (outlier) higher expression value in one out of the four experiments performed (**Figure 3D**).

To analyze whether the T cells expressing the surface markers indicative of the Th-IL-2 phenotype (CD39, CD73, CD62L) also produce IL-2 and differentially regulate the signature transcription factors, as predicted by the transcriptomic data, multicolor flow cytometry experiments were performed. These experiments revealed that allergen-specific T cell activation in the presence of BX-795 maintains a CD3^+^CD4^+^CD62L^+^ cell population of 54.2 ± 8.0% (mean ± SEM), which is barely observed upon allergen-specific T cell activation neither in the absence of BX-795 (DMSO-control) (10.1 ± 1.5%, mean ± SEM) nor in the presence of iTreg favoring factors (0.5% ± 0.2%, mean ± SEM) (**Figure S8A**). In the presence of CD39 co-expression, this cell population co-expresses IL-2 and Helios (**Figures S8C, D, G**). Notably, the CD39^+^CD62L^+^ cells generated in the presence of BX-795 contain only low levels of IFN-γ and IL-13 producing cells when compared to control cells (**Figures S8 E–F**).

**Figure 4:**
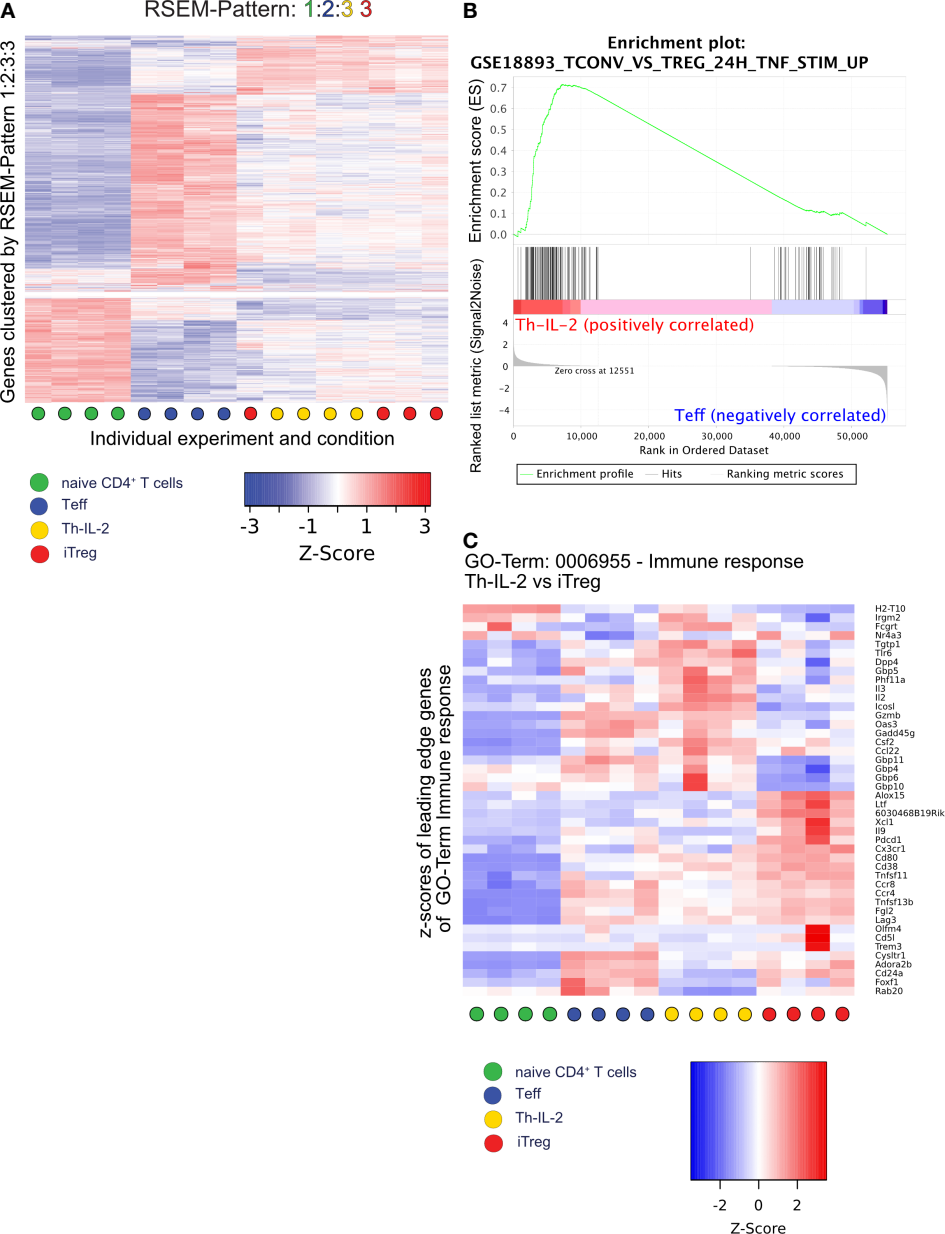
RSEM, GSEA and GO analysis identify features similar and dissimilar between Th-IL-2, iTreg and Teff. A total of 9845 protein coding genes were assigned to patterns of differential gene expression between the four experimental conditions using the rsem-run-ebseq function of the RSEM toolkit. Each gene can be part of one pattern only. Same numbers for different experimental conditions indicate similar expression of the genes in the respective pattern. Different numbers indicate significantly different gene expression between the respective experimental conditions The order of numbers assigned is shown by the condition-specific colors (indicated in the legend) on top of each heatmap/graph. **(A)**, heatmap of z-scores generated by the limma coolmap function of those 2052 genes which are similarly expressed in Th-IL-2 and iTreg cells, but have differential expression between naïve and Teff cells (pattern 1:2:3:3). **(B)**, gene set enrichment analysis (GSEA) plot of a gene set specific for Tregs after TNF-α stimulation comparing Th-IL-2 to Teff. **(C)**, heatmap showing the relative gene expression levels (z-scores) of genes identified as leading edge subset in the GO term “GO:0006955 Immune response” in an overrepresentation analysis performed by clusterProfiler/enrichGO comparing the Th-IL-2 and iTreg cell groups of samples. Based on the previous DESeq2 analysis, only genes with a log_2_-fold-change between Th-IL-2 and iTreg of >1 were selected as input for clusterProfiler/enrichGO. Sample group membership is indicated along the x-axis according to **Figure 3A**.

Subsequent assignment of mRNA expression data to patterns of similarity/dissimilarity of expression of genes using RNA-Seq by expectation maximization (RSEM) (49) revealed that the similarity between Th-IL-2 and iTreg cells was based on 2.816 out of the 9.845 protein coding genes (**Figure 4A**; **Table S2**) identified. In fact, 4.732 of the 9.845 genes are similarly regulated between Th-IL-2 and Teff cells among all differentially regulated genes detected in the experiments. However, 4.301 of these 4.732 genes are also similarly regulated in iTreg. Moreover, 163 genes are also similarly regulated in Th-IL-2 cells and naïve T cells. From that follows that only 268 genes remain which are exclusively similarly regulated in Th-IL-2 and Teff cells. This is in stark contrast to the much greater similarity of Th-IL-2 and iTreg cells. In fact, these two cell types coregulated 2.816 genes. Thus, the similarity in terms of uniquely co-regulated genes is more than 10-fold higher for Th-IL-2 and iTreg cells when compared to Th-IL-2 and Teff cells. Similar to the herein performed comparison between Th-IL-2 cells and the iTreg cells differentiated parallel gene set enrichment analyses (GSEA) confirmed the significant enrichment of genes associated with Treg cells in Th-IL-2 when compared with Teff cells (GSE18893_Tconv_vs_Treg_24h_TNF_Stim_up) (**Figure 4B**) (50). Despite the large degree of similarities, clear biological differences exist between Th-IL-2 and iTreg cells, as shown by gene ontology (GO) enrichment analyses using clusterProfiler and a list of ranked differentially expressed genes (those genes that showed a log_2_-fold change ≥ 1 between Th-IL-2 and iTreg) used as input. In fact, 54 GO terms significantly distinguished biological processes of Th-IL-2 and iTreg cells (**Table S3**). From these 54 GO terms, the term *immune response* was most significantly enriched which harbored, for example, the overexpressed *Il2*, in addition to other genes differentially expressed between Th-IL-2 and iTreg, but also *Csf2* (granulocyte monocyte colony stimulating factor 2) *Icosl* (ICOS-ligand, CD275) and *Gzmb* (granzyme B) (**Figure 4C**), the latter two representing signature molecules of most suppressive Treg (51, 52) and T regulatory 1 (Tr1) cells (53), respectively. That overexpressed IL-2 may be biologically active can be already inferred from the overexpressed *Csf2* mRNA levels. Although *Csf2* was clearly not as strongly differentially expressed in Th-IL-2 as *Il2* (average z-score for IL-2 1.4 versus 1.1 for *Csf2*) (**Figure 4C**) its increase in expression complies well with the previous finding that administration of IL-2/anti-IL-2 complexes increased *Csf2* expression (54). That the increased *Csf2* mRNA levels were not reflected by a similar tendency in soluble cytokine levels detectable in the culture supernatant of cells treated with BX-795 may be caused by the increased consumption of GM-CSF by developing antigen-presenting cells within the culture system (55, 56). As a result of the selection strategy of the genes for the GO analyses in **Figure 4C**, genes similarly or not strongly differentially expressed between Th-IL-2 and iTreg cells were not included for GO analysis. In addition, Entpd1 and Nt5e are not part of **Figure 4C**, because they are not part of the GO term *immune response*. Moreover, Th-IL-2 cells exhibited also a moderate suppressive capacity, it was, however, less pronounced compared with *in vitro* differentiated iTregs. (**Figure S9**).

### IL-2 secreted by Th-IL-2 cells is biologically active and required for the expression of the regulatory phenotype of Th-IL-2 cells

**Figure 5:**
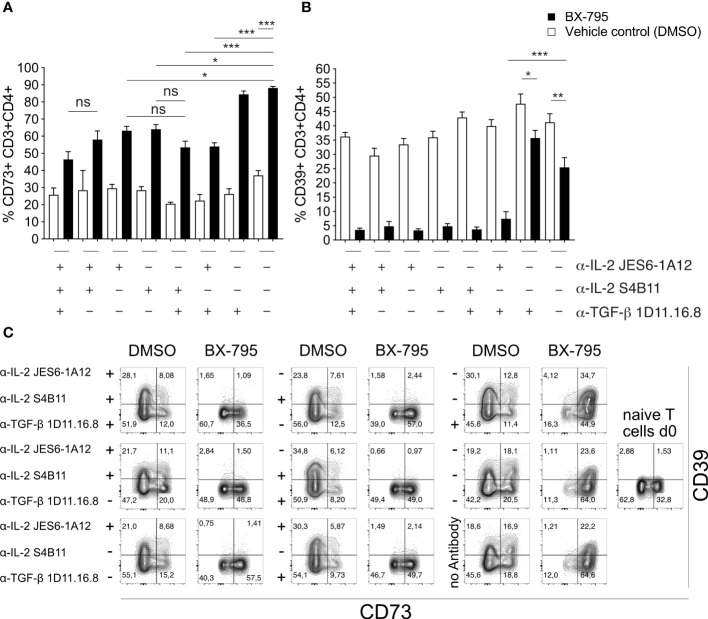
IL-2 secreted by Th-IL-2 cells is biologically active. **(A, B)**, bar graphs show the percentages of CD73^+^ (left panel) and CD39^+^ (right panel) CD3^+^CD4^+^ T cells as determined by flow-cytometry for each indicated condition. Briefly, naïve CD4^+^ T cells [1x10^5^/well] from double transgenic allergy mice were co-cultured with BMDCs [2x10^4^/well] and recombinant Art v 1 protein [1 µg/mL] in the presence or absence of BX-795 [0.24 µM]. Blocking-antibodies against IL-2 and/or TGF-β were added at the beginning of the 120 h incubation period at a final concentration of 10 µg/mL in combinations as indicated. Presence of the respective blocking antibody is indicated by a “+”, replacement of the antibody by its isotype control at a concentration of 10 µg/mL is indicated by a “-”. After 120 hours, cells were stained with fluorophore-conjugated antibodies and analyzed by flow-cytometry. **(C)**, Representative FACS plots of the experiments performed in **(A, B)** are shown. Numbers indicate percent cells in the respective quadrants. The presence or absence of blocking antibodies is indicated on the left-hand side of each set of contour plots. Data show **(A, B)**, mean values ± SEM of three independently performed experiments. **(A, B)**, one-way ANOVA with Tukey’s *post-hoc* analysis comparing each condition with each other. The p-values are indicated by *, p < 0.05; **, p < 0.01; ***, p < 0.001. ns, non-significant.

To formally demonstrate that the overexpressed IL-2 is critical for the development of the Treg-like phenotype of Th-IL-2 cells, we blocked IL-2 during BX-795-induced Th cell differentiation and monitored the expression of two *bona fide* Treg/Tr1 marker, *i.e.*, ecto-5′-nucleotidase (*Nt5e*, CD73) and ectonucleoside triphosphate diphosphohydrolase-1 (*Entpd1*, CD39), both being critically involved in adenosine metabolism (16, 46–48) which were also differentially regulated by BX-795 (**Figure 5**). Accordingly, FACS-sorted, naïve CD62L^+^CD25^-^CD44^-^CD4^+^ T cells from transgenic allergy mice were co-incubated with syngeneic BMDC in the presence of cognate Art v 1 protein in the presence or absence of BX-795 with and without IL-2 and/or TGF-β1 neutralizing antibodies for 5 days. We found that BX-795 at nanomolar concentrations IL-2-dependently but TGF-β1-independently up-regulated CD73 expression on Th-IL-2 cells (58.1 ± 5.0 versus 88.2 ± 0.9%, p = 0.0010, mean ± SEM) (**Figures 5A, B**). The other important ectoenzyme involved in adenosine generation by Treg and Tr1 cells, CD39, was by default expressed on one third of T_eff_ cells even in the absence of BX-795. Notably, CD39 expression levels on Th-IL-2 cells on day 5 were highly-dependent on the availability of IL-2, as shown by their dramatic down-regulation in the presence of neutralizing IL-2 but not TGF-β1 antibodies (**Figures 5A–C**) as assessed by multicolor flow-cytometry. Interestingly, IL-2 or TGF-β did not seem to play a direct role for the BX-795-inhibited Th2 phenotype since their individual or combined antibody-based neutralization was not able to rescue the Th-IL-2 associated inhibition of IL-4, IL-5 and IL-13 secretion (**Figure S10**) as assessed by multiplexing cell culture supernatants. That the anti-IL-2 antibodies highly effectively neutralized IL-2 can be seen by the lack of detectable IL-2 in their presence but not in their absence (**Figure S10**). Together, these results indicate that BX-795-induced IL-2 secretion is biologically relevant and contributes to the development of the Foxp3^neg^ Th-IL-2 cells.

### BX-795 administration alleviates Th2 inflammation *in vivo*

**Figure 6:**
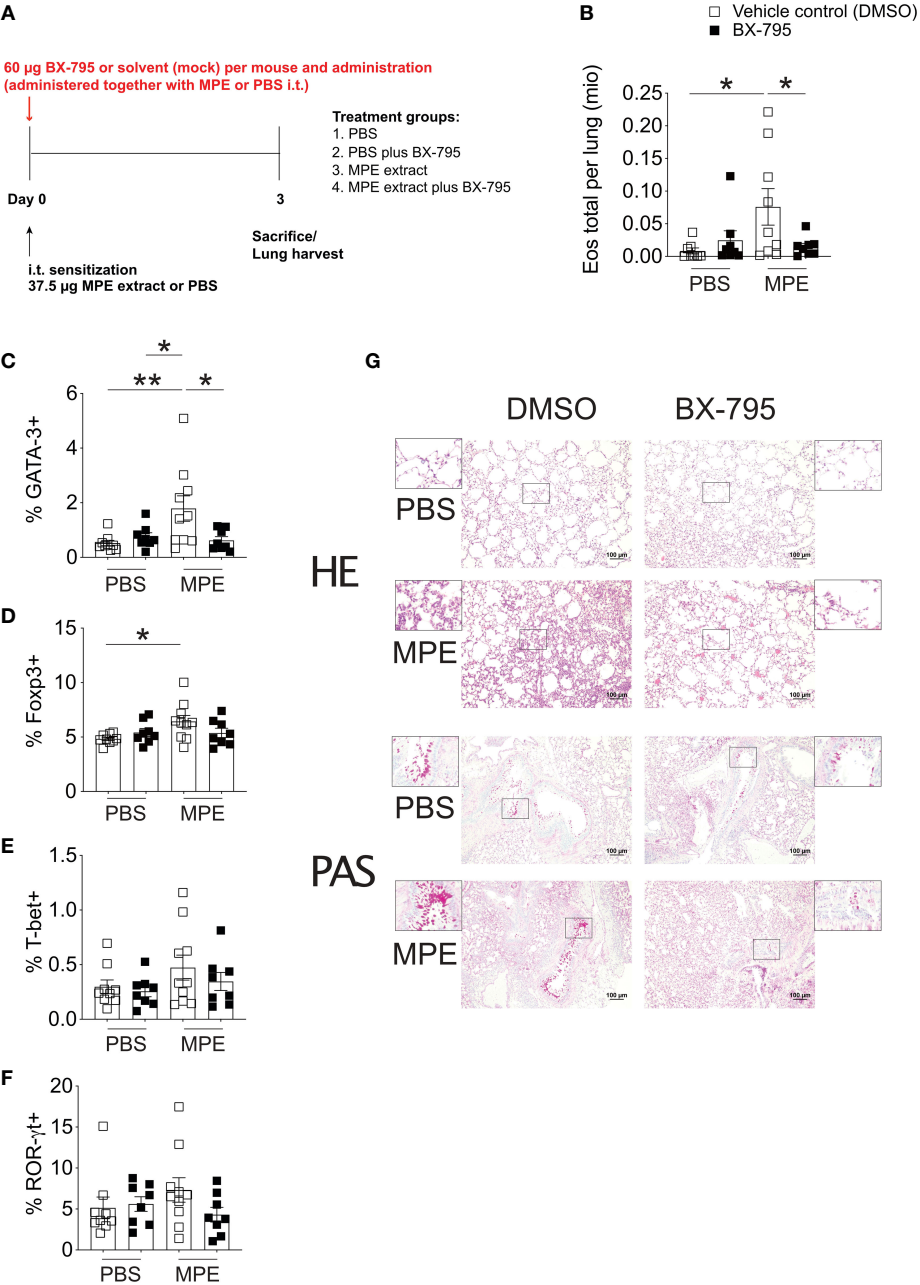
BX-795 ameliorates Th2 inflammation in a murine model of mugwort allergy. **(A)**, shown is the treatment protocol indicating the time points and dosage of mugwort-pollen extract (MPE) or sham treatment (PBS) and concomitant BX-795 exposure of mugwort-specific TCR/DR1 double transgenic C57BL/6 mice. All conditions contained the same amounts of solvent (DMSO). **(B)**, shown are the absolute numbers of eosinophils expressed as 1x10^6^ cells per lung of mice treated as in **(A)** determined by FACS analyses (gating strategy see **Figure S15**) of lung homogenates. **(C-F)**, shown are the percentages of GATA-3^+^, Foxp3^+^, T-bet^+^ and ROR-γt^+^ of CD3^+^CD4^+^ T cells in the lung of mice sensitized by i.t. administration of MPE or placebo (PBS) in the presence or absence of i.t. applied BX-795 as determined by FACS analyses. **(G)**, shown are representative lung sections stained with hematoxylin eosin (HE, upper panel) or periodic acid-Schiff (PAS, lower panel) of mice of the indicated treatment groups. **(B-F)**, data show the mean ± SEM of pooled result from two independently performed experiments containing 8-10 mice in total per group. The indicated p-values were calculated using one-way ANOVA followed by Dunnett’s test comparing MPE stimulated BX-795 treated to counterparts. Only statistically significant changes are indicated with *, p < 0.05; **, p < 0.01.

**Figure 7:**
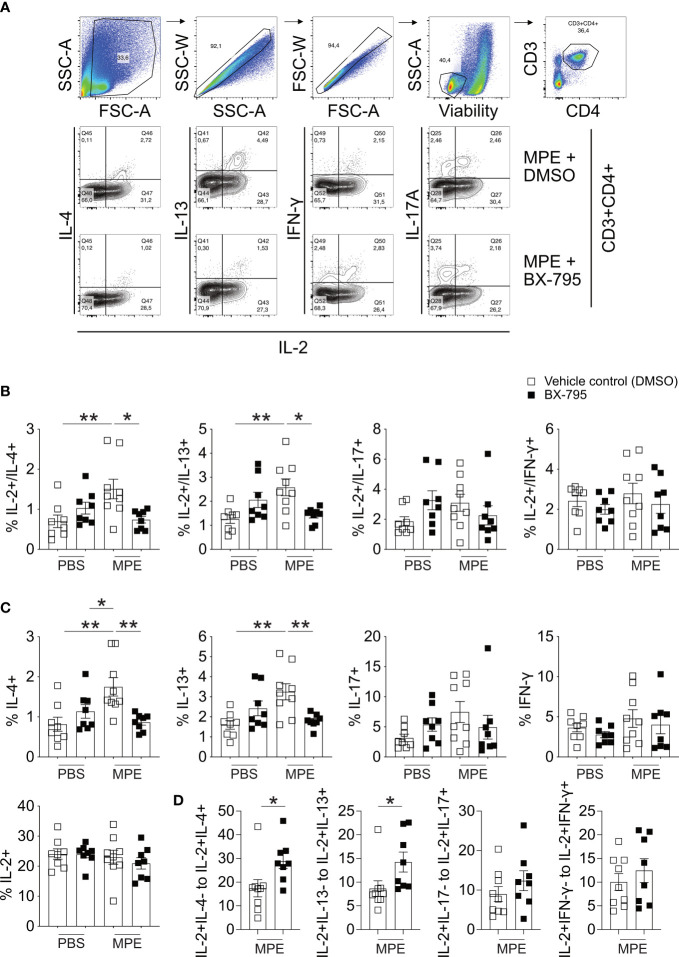
BX-795 reduces MPE induced IL-2/IL-4 and IL-2/IL-13 co-producing T cells but not overall IL-2 producers in a murine model of mugwort allergy. Lung cells derived from mice challenged as shown in **Figure 6A** were stimulated for 4 h in the presence of PMA/ionomycin and protein transport inhibitors monensin/brefeldin A and then stained for CD3, CD4 and the indicated cytokines. **(A)**, shown is the gating strategy for the analysis of IL-2/cytokine double positive cells and representative FACS plots showing co-production of indicated cytokines with IL-2 for mice treated with MPE plus solvent and MPE plus BX-795. **(B)**, shown are the percentages of cells co-producing IL-2 and the indicated second cytokine as percent of CD3^+^CD4^+^ T cells for each treatment group. **(C)**, shown are the percentages of all cells of CD3^+^CD4^+^ T cells producing the indicated cytokine. The total percentage of the respective cytokine producing cells was calculated from the IL-2/cytokine gates shown in **(A)**. The percentage of all IL-2 producing cells of CD3^+^CD4^+^ T cells was calculated from the IL-2/IL-4 plots. **(D)**, shown are the ratios of IL-2^+^/Cytokine^-^ cells relative to IL-2^+^/Cytokine^+^ cells for MPE plus solvent and MPE plus BX-795 treated mice. Cells stained for the indicated cytokines were stimulated for 4 h in the presence of PMA/ionomycin and protein transport inhibitors monensin/brefeldin A, Data show the mean ± SEM of pooled results from two independently performed experiments containing *n = 8* both PBS groups, MPE plus BX-795 and *n = 9* MPE plus DMSO **(B-D)**. The indicated p-values were calculated using one-way ANOVA followed by Dunnett’s test **(B, C)** or by an unpaired two-sided Student’s t-test **(D)**. All comparisons performed against MPE plus solvent treated. Only statistically significant changes are indicated with *, p < 0.05; **, p < 0.01.

To translate our findings to preclinical models of allergic airway inflammation, recently established mugwort allergy mice (35) were challenged with mugwort pollen extract (MPE) in the presence and absence of the Th-IL-2 inducer BX-795 (**Figures 6**, **7**). *In vivo* infiltration of the lungs with effector cells and the resulting lung histology were determined. While application of vehicle control (DMSO) or BX-795 *per se* did not change numbers of eosinophils, MPE challenge increased eosinophil numbers significantly, which was completely abrogated in MPE-challenged mice which were co-treated with BX-795 (**Figure 6B**). Similarly, the frequency of Th2 cells expressing the signature transcription factor GATA-3, and the Th2 signature cytokines IL-4 and IL-13 (**Figures 6C**; **7B, C**) were significantly reduced. In contrast, frequencies of IL-2^+^, Tbet^+^, IFN-γ^+^, ROR-γt^+^ and IL-17^+^ (**Figures 6E, F**; **7C**) T cells were comparable or only slightly different across groups. In support of our *in vitro* findings on a single cell level, the fractions of IL-2^+^ cells which co-expressed the Th2 cytokines IL-4 and IL-13, were much smaller, indicating that these cells were IL-2 producers but unable to elaborate Th2 effector cytokines (**Figure 7B**). No such effects on IL-2/IFN-γ and IL-2/IL-17 co-producing cells (**Figure 7B**) or overall IL-2^+^ CD4^+^ T cells were observed (**Figure 7C**). These findings support the previous assumption that BX-795 has a complex differentiating effect on T cells by inducing Th-IL-2 cells and does not simply drive T cells into an anergic state. Frequencies (**Figure 6D**) and numbers (**Figure S11**) of Foxp3^+^ cells were upregulated in MPE challenged mice only in the absence but not in the presence of BX-795, which was compatible with the *in vitro* finding that BX-795 inhibits Foxp3 expression in iTreg differentiation cultures (**Figures S12**, **S13** **)**. Notably, overall cellular infiltration and mucus production was reduced in MPE challenged mice treated with BX-795 as compared to sham-treated mice (**Figure 6G**). Myeloid cells other than eosinophils such as *e.g.*, neutrophils or alveolar macrophages were not altered by the drug treatment (**Figures S14**, **S15**). Experiments performed in a second model of allergic airway inflammation, based on another clinically highly relevant aeroallergen source, *i.e.*, house dust mite (HDM), gave comparable results and also revealed a distinct, albeit not significant increase of IL-2^+^ T cells (**Figure S16E**). Together these results show in two independent models of allergic airway inflammation that BX-795 treatment resulted in significant inhibition of allergen extract-induced Th2 inflammation which was paralleled by generally lower numbers of Foxp3^+^ T cells (both models) and distinctly increased IL-2^+^ cells (HDM model).

## Discussion

Based on previous findings highlighting the importance of IL-2 for Treg expansion *in vivo* (8, 9), we hypothesized that SMI inducing IL-2 hypersecretion by T cells activated *via* their TCR would lead to the differentiation of cells with a Treg phenotype, which may have implications for the treatment of allergic inflammation. Indeed, we identified here a previously unrecognized function of the multikinase PDK-1/IKKε/TBK-1 small molecule inhibitor BX-795, namely its capability of inducing an IL-2 hypersecreting Th cell type upon TCR activation. This was intriguing because the other SMI studied, particularly the previously described TBK1 inhibitor amlexanox, failed to do so (43). Our initial screen was performed in Jurkat E6-1 cells, because these cells historically formed one of the centerpieces for the detailed investigation of T cell receptor signaling processes. While human HPB-ALL and HuT-78 and murine EL4 and LBRM-33 cells belonged to the “legendary group” of immortalized T cell lines, Jurkat E6-1 T cells stood out (26). The merits of Jurkat E6-1 T cells as *in vitro* test system for TCR-signaling are several-fold: i) they are excellent IL-2 producers upon TCR ligation (27); ii) they produce IL-2 especially well when two signals are provided to them for stimulation, one of them being a TCR-derived signal the second one a signal activating PKC, such as phorbol myristate acetate (57); iii) they were the basis for a number of sublines, generated by somatic mutation and lacking, for instance, TCR αβ (58), CD3 (59) and CD45 (60); iv) TCR-mutant Jurkat E6-1 cells were the basis for showing that a calcium signal, *e.g.*, provided by a calcium ionophore, could bypass the TCR-signal-requirement and lead to full-blown T cell activation in the presence of PKC activation (57); v) Jurkat E6-1 T cells were the first T cells to be used in fluorescent calcium measurements by *Imboden et al.* (59).; vi) these cells were the basis for the description of protein tyrosine kinase signaling and thus spearheading the characterization of LCK, ZAP70, CARMA1, and a number of further signaling molecules involved in the TCR-signaling pathway (61–63)); vii) nowadays, Jurkat E6-1 cells form the basis for genetic and functional (reporter) screens devoted to human T cell and HIV biology, among other topics (64–67). The fact, that a currently performed data-base search for this cell line revealed 400 citations during the year 2022 in PubMed indicates that this immortalized human T cell line, since its establishment more than 40 years ago, is still a very valuable tool for immunologists world-wide.

Hypersecreted IL-2 was identified upon activation of both human and murine T cells stimulated by surrogate TCR/CD3 ligation (OKT3), superantigen from *Staphylococcus aureus* or nominal antigen (major mugwort pollen allergen, Art v 1). Although IL-2 hypersecreting Th-IL-2 cells lacked Foxp3 expression on the mRNA and protein level, they were found to mimick iTreg by mRNA expression phenotype. In fact, RNA-Seq analyses identified 2.816 protein-coding genes which expression was similar in Th-IL-2 and iTreg cells but exhibited differential regulation in both naive and effector CD4^+^ T cells. For comparison, only 268 genes are coregulated in Th-IL-2 cells and Teff cells, indicating the much lower degree of relatedness between these two cell types. The hypersecreted IL-2 was found to be biologically relevant, as its antibody-based neutralization, *e.g.*, completely inhibited the activation-induced up-regulation of the Treg signature surface molecule ecto-5′-nucleotidase (*Nt5e*, CD73), which is well-known for its triggering of the degradation of AMP towards regulatory adenosine (68). In contrast, the increased IL-2 secretion levels were not required for the Th2 inhibitory capacity of BX-795 as shown in experiments using blocking antibodies (**Figure S10**). The suppressive potential of BX-795-induced T cells, although somewhat lower when compared to iTregs (**Figure S9**), is in line with previous reports showing that PDK1 activity contributes to the suppressive capacity of regulatory T cells (69).

Upon allergen-specific challenge in the presence of BX-795, T helper cells downregulated Th2 immunity *in vivo* and made mice less susceptible for the development of allergic airway inflammation. This was mirrored in T cells by the significant downregulation of Th2 cytokine production and the expression of the Th2 signature transcription factor GATA-3, which resulted in a reduced pathological influx of eosinophils into the lungs of allergen-sensitized and -challenged mice (**Figures 6B**; **S16B**). While Th2 cell-based reduction of cytokine production certainly contributed to reduced eosinophil infiltration, we cannot exclude that direct effects on innate immune responses contributed to the observed reduction in eosinophils. For instance, it was recently shown that cGAMP triggered HDM extract-induced asthma in mice *via* IL-33 in a TBK-1 dependent manner which could be antagonized by the *bona fide* TBK-1 inhibitor amlexanox (70).

We initially hypothesized that Th-IL-2 cells may be generated by modulation of Signal-1 strength. Indeed, when looking at TCR-induced proliferation in allergen-specific T cells, we observed that BX-795 moderately inhibited T cell proliferation (**Figure S5**). Also, TCR-induced intracellular Ca^2+^-fluxing in Jurkat E6-1 and primary human T cells as determined by the calcium indicator Indo-1 was slightly reduced at biologically relevant BX-795 concentrations (**Figures S2A, B**). These findings may be related to early BX-795-attenuated tyrosine-phosphorylation (as determined with the anti-pan tyrosine-phosphorylation antibody 4G10) (**Figure S2**). However, BX-795 sustained overall (**Figure S2C**), Lck- (**Figure S2D**) and CD3 ζ-chain-specific (**Figure S2E**) tyrosine-phosphorylation at later time points, while it left Erk tyrosine-phosphorylation unaffected (**Figure S2F**) when compared to stimulation with solvent conditions (DMSO). These findings let us speculate that BX-795 may enhance IL-2 expression by disinhibiting a TCR-distal feedback loop, which may explain how, in fact, attenuated Signal-1 strength increases IL-2 production. Such a feedback loop had indeed been posited for IKKε, suggesting that the BX-795 target IKKε provided a negative TCR-signaling feedback (45). In that study, knockout of IKKε resulted in decreased phosphorylation of the transcription factor NFATc1 and enhanced its nuclear translocation thereby enhancing IL-2 expression (45). However, when we investigated NFATc1 translocation by imaging flow cytometry in the presence of BX-795, we did not find evidence for enhanced NFATc1 translocation. Rather, in our studies, the increase in IL-2 secretion clearly preceded the expression and translocation of NFATc1 (**Figure S4**), ruling out direct involvement of the IKKε-NFATc1 axis in the IL-2 hypersecretion phenotype, and suggesting a hitherto undisclosed mechanism. That the activation induced shut-off of IL-2 may depend on a suppressor, which is sensitive to cycloheximide has been shown previously and allows to speculate that this yet unidentified suppressor could be a target of BX-795 (71). The authors cannot completely exclude the possibility of decreased consumption of secreted IL-2 as a possible contributor to the elevated IL-2 levels detectable in culture supernatants, *e.g.*, due to decreased CD25 expression. However, it seems rather unlikely that this is the only factor for the increased IL-2 levels in culture supernatants, because IL-2 mRNA expression levels were also significantly increased in human (**Figure 1**) and murine T cells (**Figure 3**). In addition, increased IL-2 levels were also observed at the single cell level in flow cytometry (**Figure S8**), suggesting that increased production of IL-2 by Th-IL-2 cells is certainly an important, if not the main, factor for the observed increase in IL-2 levels in supernatants.

RNA-Seq experiments showed that BX-795-induced Th cells appeared to be way more similar to iTregs than to naïve T cells or Teff (**Figure 3C**). This indicated that BX-795 does not inhibit the transition from naïve T cells towards a differentiated phenotype but rather directly induces a distinct Th cell phenotype which is highly similar to iTregs which we refer to here as Th-IL-2 cells. Although there was a high degree of similarity between Th-IL-2 cells and iTreg, Th-IL-2 cells completely lacked Foxp3 expression. Moreover, under iTreg differentiating conditions BX-795 reduced numbers of CD25^+^Foxp3^+^ Tregs *in vitro* from 50.4 ± 7.5% mean ± SEM to 19.2 ± 4.0% mean ± SEM, corresponding to a 62% decrease (**Figures S12**, **S13**). In contrast, the ecto-5’-nucleotidase CD73 and the ectonucleoside triphosphate diphosphohydrolase-1(CD39), both important adenosine-generating enzymes of Treg cells (48, 72), remained highly expressed (98.2 ± 0.7 mean ± SEM versus 96.5 ± 1.6% mean ± SEM) or were only modestly downregulated (17.56 ± 4,2% mean ± SEM versus 11.99 ± 4.8% mean ± SEM) (**Figures S12**, **S13**). However, another transcription factor closely related to the development of Treg cells, Ikzf2 (Helios) remained stably expressed in iTregs even in the presence of BX-795. This was in concordance with our finding that Helios expression was significantly induced in the larger part of Th-IL-2 cells (**Figures 3D**; **S8D**), and may indicate that the BX-795 targeted pathway represents a switch for the differentiation of Helios^+^/Foxp3^-^ T helper cells (73). Although differences between Foxp3^+^/Helios^+^ T cells and Foxp3^+^/Helios^-^ have been associated with different types of regulatory T cells the phenotypic characteristics of Helios^+^/Foxp3^-^ T cells have not been described in detail so far (46, 47). Helios was proposed as a specific marker for thymic-derived Treg cells (47) and the co-expression of Helios and Foxp3 was reported to increase the regulatory activity of human engineered regulatory T cells (74). Moreover, Helios expression has been linked to the control of several regulatory functions of Treg cells (46) and has been described as an exclusive marker of activated Treg cells expressing the markers GARP/LAP (75). We could confirm our findings obtained in the RNA-Seq experiments on the protein level in flow cytometric analyses demonstrating that BX-795-induced expression of the transcription factor Helios in a fraction of Th-IL-2 cells (**Figure S8**). Notably, Helios expression was independent of soluble IL-2 and TGF-β levels as determined with blocking antibodies (data not shown). Whether Helios is responsible for the induction of the here presented T cell phenotype remains to be shown. The BX-795-induced increase in Th-IL-2 cells amounted to 36.1 ± 6.2% (BX 1.2 µM, % IL-2^+^ of CD3^+^CD4^+^, mean ± SEM) while only 12.8 ± 1.2% (% of CD3^+^CD4^+^IL-2^+^, mean ± SEM) of Th-IL-2 cells were co-expressing Helios on a single cell level. This may indicate that Helios positivity is either a salient feature of a subset of Th-IL-2 cells. Alternatively, parallel immunological detection of Helios and IL-2, which is technically difficult because of gross differences in the optimal composition of the fixation reagents used, could considerably underestimate the number of Th-IL-2 cells co-expressing Helios as determined by flow cytometry. A regulatory cell type lacking Foxp3 expression is represented by IL-10^+^Foxp3^-^ Treg cells (76). Since treatment with BX-795 decreased the number of IL-10^+^ T cells, it seems rather unlikely that Th-IL-2 cells resemble IL-10^+^Foxp3^-^ Treg cells. However, the authors cannot exclude the possibility that Th-IL-2 cells share a common progenitor with IL-10^+^Foxp3^-^ cells.

Apart from the induction of Th-IL-2 cells resembling iTregs, the inhibition by BX-795 of type 2 immunity was the second major finding of this study. In fact, BX-795-dependent inhibition of type 2 cytokine secretion was a robust trait observed in different species and model systems *in vitro* and *in vivo* applying, among others, double transgenic allergy mice (**Figures 6**, **7**) and wild type C57BL/6 mice for detailed preclinical analyses (**Figure S16**), which was accompanied by a decrease in GATA-3 expression in lung T cells of allergen-sensitized and challenged mice (**Figures 6**; **S16**). Moreover, the influx of eosinophils into the lungs of allergen-exposed mice was reduced to background levels in the presence of BX-795, as was the formation of goblet cells, indicating the far-reaching effects of blockade of type 2 immunity by BX-795. Interestingly, GM-CSF which is a cytokine also secreted by Th2 cells was not inhibited throughout our studies in different model systems (77). This raises the question whether Th-IL-2 cells have similarities with the recently described T_GM_ cells (78). The transcription factors c-Maf and Fli-1 have been described as regulators of GM-CSF expression (79, 80). Previously, the transcription factor c-Maf has been shown to be exclusively involved in positive regulation of IL-4 transcription but not other Th2 cytokines (81). In our transcriptomic analyses, up-regulation of c-Maf was clearly inhibited by BX-795 whereas Fli-1 was increased although non-significantly (**Figure S17**). This may explain the discrepancy observed with regards to IL-4 inhibition but GM-CSF maintenance. Somewhat similar to GM-CSF, also IL-17A was not inhibited throughout our studies by BX-795. Th17 cell differentiation has been shown to rely on the cell cycle-dependent function of CARMA1, which suggests that the changes in cytokine expression seen in Th-IL-2 cells are not explainable by mere inhibition of cell cycle progression/proliferation as inhibition of IL-17 expression would then also be an expected outcome (82).

This study is not without certain limitations. For instance, the epigenetic changes of IL-4, IL-5, IL-13, and IL-2 loci in Th-IL-2 cells and the stability of their phenotype over time remain to be determined in future studies using epigenetic analysis tools, *e.g., by* ATAC-Seq. Moreover, transcriptomic analyses of Th-IL-2 cells at the single cell level will help to better understand the transition processes that drive naïve T cells to develop into Th-IL-2 cells and will be necessary to identify and compare phenotypic clusters of *in vivo*-induced with *in vitro*-generated Th-IL-2 cells.

Furthermore, we have not yet answered the question why the IL-2 hypersecreting phenotype, which was so robustly reproducible *in vitro* with primary (**Figures 2**; **S3**) but also with Th2-differentiated (**Figure S6**) cells, was not more clearly apparent in the allergic airway inflammation models *in vivo*. Our findings in that respect may be attributable to variables such as available drug concentration over time, complexity of the tissue/cellular composition, different pharmacokinetic and pharmacodynamic parameters of BX-795 or IL-2 itself, which are less well controllable and assessable *in vivo* when compared to *in vitro* experiments. Nevertheless, frequencies of IL-2^+^/IL-4^+^ and IL-2^+^/IL-13^+^ T cells were clearly reduced *in vivo* (2.6 ± 0.4% and 1.5 ± 0.3% to 1.4 ± 0.1% and 0.7 ± 0.1% mean ± SEM, respectively) while overall IL-2 producing cells remained the same (**Figure 6**). It is tempting to speculate that the cell fractions turning into IL-2 single positive cell types could be identical to the cell type observed *in vitro* upon BX-795 stimulation. In addition, our study is limited by the fact that we focused primarily on the effects of BX-795 on the differentiation and function of CD4^+^ T helper cells. Thus, we can only speculate how BX-795 may act on other cell types such as, *e.g.*, CD8^+^ T cells, dendritic cells or type 2 innate lymphoid cells (ILCs), the latter representing recently discovered key players in the initial phase of allergic diseases (83).

Also, the role and capability of Th-IL-2 cells to regulate levels of immunosuppressive adenosine by virtue of their CD73 and CD39 up-regulation requires to be determined in future studies.

Taken together, we have provided evidence in this study of the potential of BX-795 to limit Th2 inflammation and inhibit T cell activation and proliferation in a dose-dependent manner in multiple species and model systems. If applied at a constant (low) dose over time during differentiation of naïve T cells, BX-795 induced a phenotype displaying characteristics of iTregs with hallmark differences between Th-IL-2 cells and iTregs seen with regards to IL-2 secretion and Foxp3 expression. In addition to other studies showing anti-inflammatory capacities of BX-795 (84) and anti-viral effects (85) we here extend the potential field of application for this SMI compound towards Th2 inflammatory diseases such allergic asthma.

Since the transcriptional regulation of type 2 cytokine expression in ILC2 cells has been found to be very similar to that in Th2 cells, it can be speculated that BX-795 treatment would produce similar effects, *i.e.*, inhibition of type 2 inflammatory cytokine secretion, in this cell type (86).

## Data availability statement

The RNA-seq data presented in the study are deposited in the GEO repository, accession number GSE224271 and are accessible under the following link: https://www.ncbi.nlm.nih.gov/geo/query/acc.cgi?acc=GSE224271.

## Ethics statement

The studies involving human participants were reviewed and approved by Ethics Commission of the Medical University of Vienna, Borschkegasse 8b/E06, 1090 Vienna, Austria (EK Nr: 203 1565/2017). The patients/participants provided their written informed consent to participate in this study. The animal study was reviewed and approved by Institutional Review Board of the Medical University of Vienna and the Federal Ministry of Science, Research and Economy, Minoritenplatz 5, 1010 Vienna, Austria (GZ : BMBWF-66.009/0288-V/3b/2018).

## Author contributions

PT and WP conceived the research; PT designed, performed and analyzed most of the experiments with the help of BK, US, CK, PSc, DT, and MZ; LR, JK, and TB helped performing and analyzing Imaging flow-cytometry experiments. TB provided resources and infrastructure. AN generated double transgenic allergy mice. SJ and PSt provided reagents and materials. GG provided recombinant Art v 1 protein. HS provided resources and ideas. SD performed bioinformatic analyses. PT and WP wrote the paper. All authors contributed to the article and approved the submitted version.
