# Supplementary material for: The small molecule inhibitor BX-795 uncouples IL-2 production from inhibition of Th2 inflammation and induces CD4+ T cells resembling iTreg
Source: Front Immunol. 2023 Apr 6;14:1094694. doi: 10.3389/fimmu.2023.1094694 (PMC10117943; doi:10.3389/fimmu.2023.1094694)
Supplement: Supplementary file 1 [file DataSheet_1.docx]

**SUPPLEMENTAL INFORMATION FOR ONLINE SUPPLEMENT**

**The small molecule inhibitor BX-795 uncouples IL-2 production from inhibition of Th2 inflammation and induces CD4^+^ T cells resembling iTreg**

**Running title: BX-795 alleviates allergic inflammation**

Peter A. Tauber^1^ Bernhard Kratzer^1^, Philipp Schatzlmaier^2^, Ursula Smole^1^, Cordula Köhler^1^, Lisa Rausch^3^, Jan Kranich^3^, Doris Trapin^1^, Alina Neunkirchner^1^, Maja Zabel^1^, Sabrina Jutz^1^, Peter Steinberger^1^, Gabriele Gadermaier^5^, Thomas Brocker^3^, Hannes Stockinger^2^, Sophia Derdak^4^ and Winfried F. Pickl^1,6^

^1^ Medical University of Vienna, Center for Pathophysiology, Infectiology and Immunology, Institute of Immunology, Lazarettgasse 19, 1090 Vienna, Austria

^2^ Medical University of Vienna, Center for Pathophysiology, Infectiology and Immunology, Institute for Hygiene and Applied Immunology, Kinderspitalgasse 15, 1090 Vienna, Austria

^3^ Ludwig Maximilian University (LMU) Munich, Institute for Immunology, Biomedical Center (BMC), Faculty of Medicine, Munich, Germany

^4^ Medical University of Vienna, Core Facilities, Lazarettgasse 14, 1090 Vienna, Austria

^5^ University of Salzburg, Department of Biosciences, Salzburg, Austria

^6^ Karl Landsteiner University of Healthcare, Krems, Austria

**Corresponding author:**

Winfried F. Pickl, MD

Medical University of Vienna, Center for Pathophysiology, Infectiology and Immunology, Institute of Immunology, Lazarettgasse 19, 1090 Vienna, Austria.

Phone: (+431) 40160 33245.

Fax: (+431) 40160 933245.

Email: [winfried.pickl@meduniwien.ac.at](mailto:winfried.pickl@meduniwien.ac.at).

ORCID ID: orcid.org/0000-0003-0430-4952

**SUPPLEMENTAL MATERIALS AND METHODS**

**Stimulation of Jurkat E6-1 T cells for cytokine production**

Jurkat E6-1 cells (ATCC, Manassas, VA, USA) cultured at a cell density allowing logarithmic growth (< 1.5x10^6^/mL) were stimulated in 96-well flat bottom plates (Sarstedt, Nürmbrecht, Germany) at a final concentration of 1x10^5^ cells/well in a total volume of 200 µL. As medium, RPMI-1640 (GE Healthcare, Boston, MA, USA) supplemented with 10 % FCS, penicillin, streptomycin and amphotericin (Thermo Fisher, Waltham, MA, USA) and L-Glutamine (GE Healthcare, Boston, MA, USA) was used. For treatment of cells, DMSO (Sigma-Aldrich, St. Louis, MO) or the indicated inhibitors (see **TABLE S1** for details) dissolved in DMSO were used. For stimulation of cells, plates were coated with 100 µL of 1 µg/mL CD3 antibody (OKT3, Janssen-Cilag GmbH, Neuss, Germany) at 4°C in DPBS with Ca^2+^/Mg^2+^ overnight (GE Healthcare, Boston, MA, USA). Plates were washed two times with 100 µL DPBS with Ca^2+^/Mg^2+^ (GE Healthcare, Boston, MA, USA) before cells and medium with DMSO or targeted drugs were added. After harvesting of 150 µL of the respective supernatants for subsequent cytokine analyses remaining cells were assessed for viability by flow cytometry staining for dead/apoptotic cells with PI/Annexin-V. Accordingly, cells were washed twice with DPBS w/o Ca^2+^/Mg^2+^ and then resuspended in Annexin-V staining buffer (0.01 M HEPES, 2.5 mM CaCl_2_, 0.01 M NaCl, pH adjusted to 7.4 with NaOH) containing 1:100 diluted Annexin-V FITC (Biolegend, San Diego, CA, USA) and 1:1000 propidium-iodide (Becton Dickinson, San Diego, CA, USA) and assessed immediately on a BD Fortessa flow cytometer as described [1].

**IL-2 RT-qPCR analysis**

Jurkat E6-1 cells were seeded in 48-well plates (Sarstedt) at a concentration of 1x10^6^ cells/well and stimulated with 1x10^6^ CD3/CD28 coated Dynabeads (Thermo Fisher Scientific) in a final volume of 400 µL at 37°C, 5 % CO_2_ for 6 hours. Subsequently, plates were centrifuged (500 g, 5 minutes), supernatants were withdrawn and cells lysed in 500 µL/well TRI-reagent (Sigma-Aldrich) by re-suspending the cells five times. Cell lysates were transferred into 1.5 mL microcentrifuge tubes (Eppendorf, Hamburg, Germany) and 100 µL of 1-bromo-3-chloropropan (Sigma-Aldrich) was added to each tube followed by vortexing for 10 seconds, after that lysates were rested at room temperature for 10 minutes. Afterwards, tubes were centrifuged (12,000 g, 2°C, 15 minutes) and 150 µL of the upper aqueous phases were transferred to new 1.5 mL tubes for further processing of mRNA. To the aqueous phases, 0.5 mL of 2-propanol were added, tubes were vortexed for 10 seconds; afterwards the lysates were rested at room temperature for 10 minutes. Then, the tubes were centrifuged at 4°C at 12,000 g for 5 minutes. Supernatants were carefully discarded, and RNA pellets were resuspended in 1 mL of 70 % ethanol followed by vortexing. Then, tubes were centrifuged (2°C, 7,500 g, 5 minutes) and supernatants were discarded and RNA pellets dried in tilted tubes at room temperature over sterile kimwipes for 10 minutes. Subsequently, the almost dry pellet was resuspended in 30 µL of DNAse/RNAse-free H_2_0 and RNA concentrations were determined on a NanoDrop 2000 analyzer (Thermo Fisher Scientific). For reverse transcription of RNA into DNA, 1 µg of isolated RNA was used in a final reaction volume of 20 µL. Reverse transcription was performed with the MMuLV reverse transcriptase (New England Biolabs, Ipswich, MA, USA), in the presence of 2 mM dNTPs (Thermo Fisher Scientific) and oligo d(T)_23_ VN primers (NEB) according to the manufacturer’s recommendations. After generation of cDNA, 30 µL of DNAse/RNAase free H_2_0 was added to each PCR tube and cDNA samples were stored at -20°C. Real-time quantitative (q)PCR was performed on a C1000 ThermalCycler (Biorad, Hercules, CA, USA) using 2 µL of cDNA, 5 µl SYBR green mix (Biorad) and 500 nM forward and reverse primers for IL-2, IFN-γ and β2M (see **TABLE** **S4** for sequences) in a final volume of 10 µL using the following conditions; initial denaturation at 95°C for 3 minutes; followed by 39 cycles of denaturation at 95°C for 10 seconds, annealing/extension at 60°C for 30 seconds. Alternatively, in some experiments RNA isolation was performed using the RNeasy Mini Kit (Qiagen) followed by reverse transcription using the LunaScript RT SuperMix Kit (NEB) and qPCR was performed using the Luna Universal qPCR Master Mix (NEB) with the same cycling conditions as described above. Melting curves were run after the final cycle at 65°C for 5 seconds and at 95°C for 30 seconds. Relative IL-2 gene expression levels were normalized to those of the housekeeping gene β2-microglobulin [2]. Only samples with clear-cut product-specific melting curves were used for further analyses.

**Generation of Jurkat E6-1 cell lysates for detection of protein phosphorylation by Western blotting**

Jurkat E6-1 cells (ATCC) were cultured in RPMI-1640 supplemented with 5 mM L-glutamine, 10 % FCS (GIBCO, Thermo Fisher Scientific) and antibiotics/antimycotics (penicillin, streptomycin and amphotericin). One day prior to activation and lysate preparation, cells were split, re-suspended in fresh medium containing only 1 % FCS and thus starved at a cell density of 5x10^5^ cells/mL overnight. Prior to stimulation and lysate preparation, cells were adjusted to a concentration of 3x10^7^ cells/mL in plain RPMI-1640 after being washed once with plain RPMI-1640. Fifty microliter aliquots of this cell suspension (1.5x10^6^ cells) were transferred into 1.5 mL microcentrifuge tubes and 50 µL of plain RPMI-1640 containing DMSO or BX-795 was added. The final concentration was 0.25 % v/v DMSO for all conditions with or without BX-795 (0.5 µM, 6 µM or 30 µM). Cells were then rested in a 37°C water-bath for 90 minutes. Afterwards, cells were incubated on ice for 10 minutes and 3.5 µL of the anti-TCR-beta mAb C305 (hybridoma supernatant, generously provided by Dr. Arthur Weiss, UCSF, CA, USA [3]) diluted 1:10 in DPBS containing Ca^2+^/+Mg^2+^ were added to each sample except for the pervanadate (positive) control sample. The positive control sample was supplemented with 10 µl of pervanadate solution consisting of 90 µL sodium-orthovanadate (1 mM) in H_2_O plus 10 µL of H_2_O_2_ (30 % w/v), mixed, and incubated in the dark for 10 minutes prior to use. All microcentrifuge tubes were pulse vortexed for less than 1 second and then put back on ice for 3 minutes. Subsequently, all samples were incubated in a 37°C water bath. After 1, 3, 5, 10 and 30 minutes, the respective samples were removed from the water bath and put onto ice. Then, cell samples were re-suspended with 500 µL of ice-cold stop-buffer (20 mM TRIS-HCl, 140 mM NaCl, 0.1 mM sodium orthovanadate) and inverted once. Then, tubes were spun in a precooled microcentrifuge at 500 g and 4°C for 30 seconds. Supernatants were carefully withdrawn using first 1 mL and then 200 µL pipettes respectively. Pellets were then re-suspended in 75 µL ice-cold lysis buffer (stop-buffer containing 1% NP-40, 20 mM NaF, 1% Halt-protease inhibitor (Thermo Fisher Scientific)) by re-suspending 3 times. Tubes were then snap-frozen in liquid nitrogen and stored at -80°C. For determination of protein concentrations, lysates were centrifuged in a pre-chilled microcentrifuge (4°C) at 21.000 g for 5 minutes, and supernatants subjected to the micro BCA protein assay kit (Thermo Fisher Scientific) according to the manufacturer’s recommendations. Subsequently, lysates were adjusted to the lowest concentration determined in the respective experimental set of lysates by adding lysis buffer. Fifteen microliters of each lysate was mixed with 5 µL of 4 x reducing SDS sample buffer (Tris base 200 mM, DTT 300 mM adjusted to pH = 6.8 with HCl, SDS 4 % (w/v), glycerol 40 % (w/v), bromophenol blue 0.04 % (w/v) stored at -20°C) and boiled at 99°C in a microcentrifuge heater (Eppendorf thermomixer C) for 5 minutes. Afterwards, samples were subjected to SDS-PAGE (4 % stacking gel, 10 % running gel). Electrophoresis was performed in ELFO-buffer prepared as 10x stock solution: 250 mM Tris base, 1.9 M Glycine, 1 % SDS (w/v) and used at 1x supplemented with 0.1 mM orthovanadate) at a fixed voltage of 90 V for 15 minutes followed by 120 V for approximately 90 minutes (Peq-Lab, VWR, Erlangen, Germany) on a Peq-Lab apparatus. Separated proteins were blotted onto PVDF membranes (Merck-Millipore, Darmstadt, Germany) using a semi-dry Western blotting system (Peq-Lab, VWR). Membranes were first incubated in pure ethanol or methanol for 5 minutes and then transferred to Western blot buffer for 5 minutes (prepared as 10x: 250 mM Tris base, 1.9 M Glycine used as 1x in dH_2_O with 20 % (v/v) methanol). Then, the sandwich was built as follows: 3 layers of Whatman filter paper soaked in Westen blot buffer, followed by the PVDF membrane, the gel on top of the membrane and finally again 3 layers of Whatman paper soaked in Western blot buffer. Blotting was performed at a fixed current of 2 mA per cm^2^ of PVDF membrane for 1 hour. After blotting, the membranes were blocked in TBS-T buffer containing 5 % BSA (Sigma) for 30 minutes. Subsequently, membranes were cut as such that they fit into a 50 mL falcon tube and membranes were incubated under constant rotation with 1:4000 diluted 4G10 mAb conjugated to platinum HRP (Merck-Millipore) in TBS-T containing 1 % BSA at 4°C overnight. The next day, membranes were washed two times with distilled water and once in TBS-T for 5 minutes. Afterwards, membranes were rinsed twice with water, put on a piece of Parafilm (BEMIS, Sigma) and incubated with Western blot ECL substrate (Biorad) for 5 minutes before being wrapped into a waste bag foil and chemiluminescence was acquired on a FUJI LAS 4000 apparatus (Fuji, Tokyo, Japan). To perform loading controls, membranes were rinsed with water and incubated in 20 mL stripping buffer (200 mM Glycine, 3.5 mM SDS, 1 % (v/v) Tween 20, pH = 2.2 with HCl) twice for 10 minutes each and then washed 3 x in 20 mL TBS-T for 5 minutes before they were incubated with the rabbit α-human β-actin antibody (CST, Danvers, MA, USA; diluted 1:2000) in TBS-T containing 1 % BSA at 4°C overnight. On the following day, membranes were washed 3-times with 20 mL TBS-T and then incubated with a goat anti-rabbit HRP conjugated antibody (DAKO, Agilent, Santa Clara, CA, USA) at a concentration of 1:1000 in TBS-T containing 1 % BSA, at room temperature for 1 hour. The membranes were washed three times with TBS-T, incubated with ECL substrate and further processed as described above. The same protocol was applied for detection of phosphorylation of other proteins using phospho- or pan-protein specific antibodies by applying incubation times and conditions according to the manufacturers’ recommendations.

Antibodies used for Western blot analysis of protein phosphorylation with information on origin are listed in **TABLE S5**.

To quantitate signaling induction, densitometric analyses were performed using the Multigauge 3.0 software (Fujifilm, Austria). As exemplarily shown by dashed rectangles in Supplemental Figure 2 (C-F), specific signals in AU/mm^2^ were exported and corrected by background control signals. To facilitate comparison between different experiments, the maximum induction over non-stimulated baseline *(i.e.,* DMSO condition after one minute of stimulation for 4G10, pY416-Src, and CD3-zeta and after three minutes of stimulation for ERK) was set to 1 for each experiment and all other signals were normalized to the respective signal. Were applicable, paired two-tailed Student’s t-test was performed to compare induction of BX-795-incubated samples with DMSO controls.

**Intracellular Ca^2+^-fluxing experiments**

Jurkat E6-1 cells (ATCC) were cultured in RPMI-1640 supplemented with 5 mM L-glutamine, 10 % FCS (GIBCO, Thermo Fisher Scientific) and antibiotics/antimycotics (penicillin, streptomycin) and kept at a density of 5-10x10^5^ on the day before the experiment. For primary cell experiments, CD14-depleted PBMCs were thawed and kept in medium supplemented with 5% FCS. On the day of the experiment, cells were counted, washed and resuspended in a 15 mL falcon in medium incl. 1µM Indo-1 AM at a concentration of 1x10^7^/mL and incubated for 30 minutes at 37°C. Afterwards the tube was filled up with medium, centrifuged and Jurkat E6-1 and primary T cells were resuspended in medium at a concentration of 2 and 4x10^6^ cells, respectively, and split into four different falcon tubes. An equal volume of DMSO-adjusted 2x medium stocks with BX-795 was added to each tube to obtain final conc. of 0, 0.5, 6 and 30µM and cells were rested for another 30 minutes in the incubator. Cells were put on ice. To primary cells only, Beriglobin (CSL Berhing GmbH, Marburg, Hessen, Germany) was added to a final conc. of 2 % for 5 minutes, and FITC-labeled antibodies against CD14, CD16, CD19, CD56 and gamma-delta-TCR for dump staining were added according to manufacturer’s instructions for another 15 minutes. Cells were washed once with ice-cold medium and resuspended in the respective BX-795 supplemented medium at a concentration of 2x10^6^/mL. For flux measurement, 300-500 µL aliquots of labeled cells were preheated at 37°C for 5 minutes and then analyzed by flow cytometry for 1 min to record the baseline Indo-1 violet [Ca^2+^ bound] and blue [Ca^2+^ non-bound] ratio. After one minute, the anti-TCR-beta mAb C305 (kind gift from Prof. Arthur Weiss) was added (1:600 final), and after 7 minutes 4 µM ionomycin were added as ionophore positive control for another minute. Primary samples were stimulated with 4 µg/mL MEM-92, an IgM CD3 mAb, for direct crosslinking of the TCR/CD3 complex. All antibodies used for labeling and stimulation were ultra-LEAF or freshly dialyzed with DPBS, and cells were resuspended with pipette tips only without vortexing to minimize unspecific flux activation. Flux kinetics were analyzed with the Flowjo10.7.1 software, blotting the Indo-1 Violet/Blue ratio over time. To quantitate Ca^2+^-flux induction, the areas under the curve (AUC) after C305 or MEM-92 stimulation were calculated, starting at 60 seconds of recording until addition of ionomycin. Flux AUC was corrected by baseline AUC recorded during the first minute for each run to correct for variations in Indo-labeling. Signals were normalized to the condition DMSO. Were applicable, paired two-tailed Student’s t-test was performed to compare induction of BX-795-incubated samples with DMSO controls.

**Determination of cellular proliferation and cytokine production by hPBMC**

Venous blood from healthy human donors was collected after informed consent and approval by the Ethics Committee of the Medical University of Vienna (EK Nr: 1565/2017) from the cubital vein. Human PBMCs were isolated according to standard methods using Ficoll-Paque (VWR) density centrifugation. In brief, heparinized peripheral blood was diluted 1:2 v/v in IMDM medium containing supplements and containing heparin (20 units/mL, Gilvasan, Vienna, Austria). Ten milliliters of Ficoll in 30 mL tubes (Sterilin, Thermo Fischer Scientific) were carefully overlaid with 20 ml of diluted whole blood and then centrifuged without declaration at 500 g for 25 minutes. The top acellular phase was removed and the buffy coat residing on the Ficoll cushion containing the majority of PBMC was transferred into fresh 15 mL tubes using a 1 mL pipette. PBMCs were washed two times with IMDM plus supplements (1^st^ wash: 587 g, 10 minutes, 2^nd^ wash: 500 g, 5 minutes), and were re-suspended in a final volume of 1 mL and the cell concentration was determined. Stimulation of PBMC was performed in 96-well round bottom tissue culture plates (Sarstedt) at a final cell concentration of 1x10^5^ cells/well in a final volume of 200 µL AIM V medium (Gibco, Thermo Fisher scientific) containing supplements (15 µg/mL gentamicin (Gibco, Thermo Fisher scientific) and 2% human serum). Cells were either incubated in medium alone or with 50 ng/mL *Staphylococcal* enterotoxin superantigen A (SEA, Serva, Heidelberg, Germany), CD3 (OKT3, 1 µg/mL) or CD3/CD28-mAb coated beads (Dynal, 10^4^/well, Thermo Fisher) in the presence of solvent alone (DMSO) or the indicated targeted drugs at the indicated concentrations. After 72 hours of culture, plates were centrifuged at 500 g for 5 minutes and 100 µL of the supernatants were removed for subsequent cytokine analyses. Then, 100 µL of fresh AIM V medium plus supplements, containing 2 % human serum, was added to each well along with 25 µL of methyl-^3^H-thymidine (40 µCi/mL, Perkin Elmer, Boston, MA, USA) and cells were incubated at 37°C in a 5 % CO_2_ atmosphere for another 18 hours. At the end of the culture period, plates were frozen at -20°C. The next day, plates were thawed and DNA of lysed cells harvested onto filter plates using a harvester (Perkin Elmer). Then, filter plates were dried at 55°C for 1 hour, subsequently 25 µL of scintillation liquid (Perkin Elmer) was added to each well and the activity of the incorporated methyl-^3^H-thymidine determined on a MicroBeta2 2450 microplate beta-counter (Perkin Elmer).

Elaborated cytokines were determined in supernatants of the above described PBMC stimulation cultures by multiplex cytokine analyses (Luminex 100IS, Biomedica, Vienna, Austria) using the antibodies listed in **TABLE S5**.

Dynal beads were coated according to the manufacturer’s recommendations with OKT3 and CD28 mAb CD28.2 (Biolegend, San Diego, CA, USA).

**Inhibitors, peptides, MPE and HDM extract**

Immunodominant peptides from major mugwort (Art v 1_23-36_) and birch (Bet v 1_112-123_, Bet v 1_142-153_) pollen allergens were obtained from Proimmune (Oxford, ENG, UK). One milligram of lyophilized peptide was dissolved in medium containing 3 % v/v DMSO (Sigma-Aldrich, St. Louis, MI, USA) for Art v 1_23-36_ or medium containing 7 % DMSO for Bet v 1_112-123_ and Bet v 1_142-153_. *Dermatophagoides pteronyssinus* extract (Stallergenes Greer, Lenoir, NC, USA) was dissolved in an adequate volume of DPBS, containing Ca^2+^ and Mg^2+^ (Thermo Fisher Scientific, Waltham, MA, USA) to obtain a final protein concentration of 2.5 mg/mL and incubated at room temperature for 30 minutes. Dissolved and adjusted HDM extract was aliquoted and stored at -20°C until use. Mugwort pollen extract was prepared as described elsewhere [4]. Briefly, 10 g of *Artemisia vulgaris* pollen (Allergon AB, Engelholm, Sweden or Stallergens) were incubated in 100 ml of PBS (1x) by stirring at 4°C overnight. After centrifugation at 52,000 g at 4°C for 60 minutes, the supernatants were filtered and subsequently dialyzed (Spectra/Por dialysis membrane, MWCO: 6-8000, Spectrum Laboratories, Rancho Dominguez, CA) against 1 x PBS for 48 hours. The total protein concentration of the dialysate was determined by standard procedures (BCA-bicinchoninic acid protein Kit, Pierce, Rockford, IL). The lipopolysaccharide (LPS) content of the mugwort pollen extract was ≤ 0,024 U/mg. The extracts were lyophilized and aliquots were stored at –80°C until further use. Purified recombinant Art v 1.0101 and Bet v 1.0101 allergens were kindly provided by Gabriele Gadermaier (Salzburg, Austria) and Heimo Breiteneder (Vienna, Austria).

**Animal experimental procedures**

The TCR/DR1 double transgenic mouse strain, expressing a human TCR specific for the major mugwort pollen allergen Art v 1_25-36_ peptide in the context of HLA-DR1 (all back-crossed at least 10-12 times to C57BL/6J, [4] was cohoused in a conventional animal facility at the Institute of Immunology (Medical University of Vienna, Vienna, Austria). These mice were used as a source for allergen-specific T cells and antigen presenting cells and used for *in vivo* challenge experiments with mugwort-pollen extract (MPE). C57BL/6J mice were bred at the animal facility of the Medical University of Vienna and age-matched male and female mice (6-10 weeks old) were used for the house-dust mite (HDM) sensitization experiments. All mice received food and water *ad libitum*. All experimental procedures were reviewed and approved by the Institutional Review Board of the Medical University of Vienna and approved by the Federal Ministry of Science, Research and Economy (GZ:BMBWF-66.009/0288-V/3b/2018. Sentinel mice were screened for and found to be free of mouse pathogenic viruses, bacteria and parasites according to FELASA 2014 recommendations [5].

*HDM extract treatment scheme:*

*Sensitization:* Mice were sensitized intratracheally on day 0 with 30 µL DPBS w/ Ca^2^ and Mg^2+^ containing 1 µg of HDM extract protein together with plain DMSO or DMSO containing BX-795 (60 µg) (**FIGURE S16**). As controls, mice treated with DPBS w/ Ca^2^ and Mg^2+^ plus DMSO or BX-795 (60 µg) in the absence of HDM were used. The DMSO concentration was adjusted to 2% v/v in all conditions.

*Challenge:* Mice were challenged by intranasal (i.n.) administration of 30 µL of DPBS w/ Ca^2^ and Mg^2+^ containing 10 µg HDM extract protein plus plain DMSO or DMSO containing BX-795 (60 µg) on days 7, 9 and 11. Control mice received DPBS w/ Ca^2^ and Mg^2+^ alone in the presence of plain DMSO or BX-795 dissolved in DMSO. On days 8 and 10, mice were treated with DPBS w/ Ca^2^ and Mg^2+^ in the presence or absence of 10 µg HDM extract.

*MPE extract treatment scheme:*

Mice were sensitized intratracheally on day 0 with 30 µL DPBS w/ Ca^2^ and Mg^2+^ containing 36 µg of MPE extract protein together with plain DMSO or DMSO containing BX-795 (60 µg) (**FIGURE 6**). As controls, mice treated with DPBS w/ Ca^2^ and Mg^2+^ plus DMSO or BX-795 (60 µg) in the absence of MPE were used. The DMSO concentration was adjusted to 2% v/v in all conditions.

**Analyses of lung tissue**

Lungs of animals were obtained upon anatomical dissection according to standard procedures from animals sacrificed under anesthesia after exsanguination. Briefly, the post-caval lobes were transferred into cryotubes (Nunc, Thermo Fisher Scientific) and snap-frozen in liquid nitrogen and afterwards stored at -80°C for subsequent mRNA analyses. The right upper lobe was put into an embedding cassette and fixed in 4 % paraformaldehyde solution for subsequent histological processing. The remaining lung tissues were individually transferred into 70 µm nylon cell strainer (Becton Dickinson) placed into 6-well plates containing 6 mL/well of incomplete medium (RPMI-1640 medium plus a few flakes of DNAse, 10 mg/L liberase TL and PenStrepA, all reagents from Sigma Aldrich). Lungs were processed by first chopping the tissue into 5 mm pieces and afterwards mincing and sieving the tissue with the pestle of a 2 mL syringe through the cell strainer into a 50 mL falcon tube to obtain single cell suspensions. Cell strainers were washed with the medium remaining in each well and after another round of mincing, the syringe and cell strainer were washed with 10 mL of complete medium (RPMI-1640 plus 10 % FCS plus 1x penicillin/streptomycin, 5 mM L-glutamine and β-mercaptoethanol) supplemented with a few flakes of DNAse (Sigma Aldrich) into the falcon tube. Lung cell suspensions were centrifuged at 538 g (1600 rpm) for 6 minutes and erythrocytes removed by resuspension of the cell pellet in 3 mL ammonium chloride lysis buffer (155 mM ammonium chloride, 10 mM potassium hydrogen carbonate, 0.1 mM EDTA at pH 7.40, Sigma Chemicals) at room temperature for 3 minutes. Subsequently, 10 mL of complete medium was added to stop erythrocyte lysis and cells were centrifuged again at 538 g (1600 rpm) for 6 minutes, re-suspended in 4 mL complete medium and cell concentration determined using a Coulter counter (Beckmann Coulter, Brea, CA, USA) and afterwards adjusted to a cell concentration of 4x10^6^/ml in complete medium.

**Re-stimulation of lung cells and flow cytometric analyses.**

*Re-stimulation and intracellular cytokine analyses*

Lung cells were re-stimulated for intracellular cytokine analyses at a concentration of 1x10^6^ cells/mL in 6-well tissue culture plates (Sarstedt) or 96-well tissue culture plates (Sarstedt) with PMA (50 ng/mL, Sigma) and ionomycin (1 µg/mL, Sigma) in the presence of monensin (1:1000 diluted stock Invitrogen, Thermo Fisher Scientific) and brefeldin A (1:1000 diluted stock Becton Dickinson) in complete medium for 4-6 hours. After re-stimulation, cells were transferred into 15 mL falcon tubes, resuspended in 15 mL complete medium and centrifuged at 538 g (1600 rpm) for 6 minutes. Cell pellets were re-suspended in 100 µL DPBS w/ Ca^2^ and Mg^2+^ and transferred into 96-well round bottom tissue culture plates (Sarstedt). Afterwards, cells were centrifuged (500 g, 5 minutes) and supernatants were removed. Cells were washed once with 200 µL DPBS w/ Ca^2^ and Mg^2+^. Then, cells were incubated for 15 min with 100 µL of a 1:1000 dilution of Zombie Aqua fixable viability dye (Biolegend, San Diego, CA, USA) in DPBS w/ Ca^2^ and Mg^2+^. Afterwards, 100 µL of FACS buffer (PBS with 5 % BSA, 0.05 % NaN_3_ and 100mM EDTA) was added and cells centrifuged (500 g, 5 min). Then, 5 µL of anti-mouse CD16/32 antibody to block the Fc-receptors (Biolegend, San Diego, CA, USA) was added and cells incubated for 5 minutes after pulse vortexing. Afterwards, 100 µL of cell surface antibody mix (**TABLE S5**) in FACS buffer was added and cells were incubated for 30 minutes. Subsequently, 100 µL of FACS buffer was added and cells centrifuged (500 g, 5 minutes). Cells were washed once more with 200 µL of FACS buffer and then incubated with 100 µL IC Fixation buffer (Biolegend, San Diego, CA, USA) for 20-30 min. Cells were centrifuged (500 g, 5 minutes) and washed two times with 200 µL 1x Perm/Wash buffer (Biolegend, San Diego, CA, USA). After the second wash, 100 µL of 1x Perm/Wash buffer containing antibodies for staining intracellular antigens were added (**TABLE S5**). Cells were incubated for 40 minutes and then 100 µL of 1x Perm/Wash buffer was added and cells centrifuged (500 g, 5 min). Cells were washed once more with 200 µL 1x Perm/Wash and then re-suspended in 100 µL FACS buffer and centrifuged through a cell strainer into 4.5 mL FACS tubes (500 g, 5 minutes). All flow cytometric analyses were performed on a Fortessa LSR-II flow cytometer (Becton Dickinson) equipped with the Diva software and data were analyzed with the Flow Jo v 10.2. software package (Tree Star Inc., Ashland, OR, USA).

*Analysis of transcription factor activity by flow cytometry*

Lung cells adjusted to 4x10^6^ cells/mL were seeded at a concentration of 1x10^6^ cells/well in 96-well round bottom tissue culture plates (Sarstedt) and then processed as described for intracellular cytokine staining with the following modifications. For fixation, 100 µL Fix/Perm working solution from the Foxp3 transcription factor buffer set (eBioscience) were used (Thermo Fisher, Waltham, MA, USA) and for the permeabilization and staining step, 1x Perm buffer from the same staining buffer set were used.

*For exclusive surface staining* 1x10^6^ lung cells were processed in 96-well tissue culture plates and analyzed as described above for intracellular cytokines analyses.

**Assessment of cellular proliferation and cytokine production of murine splenic T cells**

*Proliferation assessment:* Single cell suspensions of splenocytes (2x10^5^/well) from double transgenic mice [4] were incubated in 96-well round bottom tissue culture plates with the indicated concentrations (0.01-22 µM) of the Art v 1_23-36_ peptide in the presence or absence of BX-795 or amlexanox (Selleckchem). Alternatively, cells were incubated in medium alone, PMA plus ionomycin, or with the irrelevant Bet v 1_142-153_ or Bet v 1_112-123_ peptides (only maximal concentration 22 µM) used as independent specificity controls in a final volume of 200 µL. For drug titration experiments, the concentrations of the Art v 1_23-36_ and Bet v 1_142-153_ peptides were adjusted to 1 µM. Cell culture supernatants were removed after 24, 48 and 72 hours for cytokine determinations. After each removal, medium was replenished with 100 µL fresh medium. After 24, 48 and 72 hours, cells were pulsed with 25 µL methyl-[^3^H]-thymidine (1 µCi/well) for 18 hours. Subsequently, plates were frozen at -20°C. The next day, DNA of cells was harvested onto filter plates with a harvester (Perkin Elmer). Filter plates were dried at 55°C for 1 hour and then 25 µL of scintillation liquid (Perkin Elmer) was added to each well and decay counted on a MicroBeta2 2450 microplate beta-counter (vendor).

*Cytokine determination assays:* Supernatants of splenocyte cultures were subjected to multiplex cytokine analyses using the Luminex system (Luminex 100IS, Biomedica, Vienna, Austria) along with the antibodies listed in Supplemental **TABLE S5**.

**Re-stimulation assays**

Splenocytes from TCR/DR1 mice were isolated as described above. Naïve CD4^+^ T cells were isolated by magnetic sorting according to the manufacturer’s instructions (Miltenyi Biotec, Bergisch Gladbach, Germany) to a routine purity of ≥ 90 %. For polarization experiments, 2x10^5^ BMDCs plus 1x10^6^ T cells were incubated in a total volume of 4 ml including 0.5 µg/ml rArt v 1 plus 10 ng/ml IL-2 (Peprotech), 10 µg/ml anti-IFN-γ (BioxCell, Lebanon, NH, USA), 5 µg/ml anti IL-12 mAb (BioXCell), 25 ng/ml IL-4 (Peprotech), to mimick Th2-skewing conditions for 72h. After this incubation time, cells were washed twice at 500 g for 5 minutes and incubated for another 72 hours in the presence of the cytokines and antibodies without the antigen. Polarization status was assessed after 72-96 hours by boosting cellular factor production by incubation of cells in the presence of PMA/Ionomycin containing additionally monensin A and brefeldin diluted according to the manufacturer’s recommendation for 4 hours. Cellular staining and subsequent flow cytometric analysis was carried out as described above and according to the intracellular-staining protocol using the antibodies listed in **TABLE S5**.

**Sorting of naïve CD4^+^ T cells**

Naïve CD4^+^ T cells were isolated from splenocytes by first performing a negative selection/enrichment with magnetic beads and LS column purification (naïve CD4^+^ T cell isolation kit, Miltenyi) according to the manufacturers’ recommendations with the following specifications. Splenocytes from double transgenic mice were smooshed through a 70 µm nylon cell strainer (Becton Dickinson) in NCM medium with the help of the pestle of a 10 ml syringe and collected by centrifugation at 500 g for 5 minutes. Subsequently, erythrocyte lysis was performed by resuspending the cell pellet in 5 mL of NH_4_Cl for 5 minutes. The reaction was stopped by adding 25 mL of NCM medium and cells were centrifuged at 500 g for 10 minutes. Then, cells were re-suspended in NCM medium and centrifuged once more at 500 g for 10 minutes. Subsequently, cells were resuspended in a PBS buffer (pH = 7.2, containing 0.5 % bovine serum albumin (BSA) and 2 mM EDTA) and incubated with a mixture of biotinylated mAbs (CD8a, CD11b, CD11c, CD19, CD45R (B220), CD49b (DX5), CD105, α-MHC Class II, Ter-119, anti TCR γ/δ) followed by incubation with anti-biotin antibody coated magnetic beads and anti-CD44 antibody coated magnetic beads and purification via LS column according to the manufacturers recommendations. After pre-enrichment, cells were stained with an antibody cocktail containing CD4, CD8a, CD25, CD44 and CD62L. Naïve T cells (CD4^+^CD62L^+^CD44^-^) were sorted using a Sony SH800 cell sorter (Sony Biotechnology, San Jose, CA, USA) by selecting lymphocytes in the FSC/SSC gate, and then gating on CD4^+^/CD8^-^ CD4^+^CD25^-^ and then CD62L^+^CD44^-^ cell types. Approximately 10 – 20 x 10^6^ cells were collected per sorting experiment, adjusted to a concentration of 4 x 10^6^/mL and used for experiments.

**Stimulation and polarization assays using FACS-sorted naïve CD4^+^ T cells with BX-795**

FACS sorted naive CD4^+^ T cells (1x10^5^/well) were stimulated with 1 µg/mL recombinant Art v 1 protein and 2x10^4^ BMDCs in 96-well round bottom well-plates for suspension cells (Sarstedt) for 5 days under Treg polarizing (iTreg; hTGF-β, 20 ng/mL, Peprotech), anti-IL-4 (5 µg/mL, BioXcell), anti-IFN-γ (5 µg/mL, BioXcell) or Th0 conditions (Teff; recombinant Art v 1 protein and BMDCs w/o polarizing reagents) with solvent or BX-795 (Th-IL-2) at the indicated concentrations (see **TABLE S6** for details on cytokines). After 5 days, supernatants were collected and cells were stained for viability, canonical Treg surface markers (CD25, CD39, CD73) and Foxp3 using the eBioscience Foxp3 transcription factor buffer set (Thermo Fisher) according to the manufacturer’s recommendations and as described above for the analyses of lung cell populations and analyzed by flow-cytometry on a BD Fortessa (Becton Dickinson). For mechanistic experiments, cells were cultured under Th0 conditions and neutralizing antibodies against TGF-β (1D11, 10 µg/mL, BioXcell) or IL-2 (S4B6-1, 10 µg/mL, BioXcell; JES6-1A12, 10 µg/mL, eBioscience) or respective isotype controls (for TGF-β: MOPC21, 10 µg/mL, BioXcell; for IL-2: RTK2758, 10 µg/mL, Biolegend) were added at a concentration of 10 µg/mL per antibody. Cells were then assessed as described above.

**T cell suppression assays**

FACS sorted naïve CD4^+^ (1 x 10^5^/well) T cells from double transgenic mice were stimulated with plate-bound CD3 [coated with 100 µL 1 µg/mL] and soluble CD28 [3 µg/mL] in the presence of BX-795 [0.24 µM and 0.48 µM] (termed “Th-IL-2 0.24” and “Th-IL-2 0.48”) or in the presence of solvent (DMSO) under iTreg polarizing conditions (TGF-β, anti-IL-4, anti-IFN-γ) (iTreg). After 120 h, cells were collected, centrifuged (500g, 5 min) and resuspended in 1mL sorting buffer (PBS, 2mM EDTA, 0.5 % FCS) containing 0.43 mmol/L Propidium-iodide (PI). Cells were sorted for FSC/SSC characteristics and PI negativity. These cells were labelled with the proliferation dye eF670 by adding 1:1000 diluted proliferation dye eF670 (ThermoFisher) in PBS w/o Ca^2+^/Mg^2+^ to the cells in the same volume of PBS w/o Ca^2+^/Mg^2+^ containing 2 % FCS under vortexing (final dilution of proliferation dye 1:2000). Cells were incubated for 7 min at 37°C and afterwards centrifuged (1000 g, 2 min) and the supernatant taken with a 1000 µL pipette. Cells were then resuspended in ice-cold NCM medium and centrifuged again (1000 g, 5 min) and adjusted to a concentration of 1x 10^6^ cells/mL in NCM medium. CD4^+^ T cells from splenocytes of double transgenic mice were enriched using the CD4^+^ T cell enrichment kit and afterwards FACS sorted for FSC/SSC characteristics and CD4^+^CD25^-^ and labelled with the proliferation dye eF670 or eF450 (Teff) as described before. T cells labelled with the proliferation dye eF670 were set together with 5 x 10^4^ of the FACS sorted CD4^+^CD25^-^ effector T cells labelled with the eF450 dye in different ratios by diluting the eF670 labelled cells (suppressor cells or controls) in NCM. Effector T cells and suppressor cells were stimulated with CD3/CD28 mouse T activator beads (ThermoFisher, 8 x 10^5^ beads per well) in 200 µL final culture volume in 96-well round bottom plates for 96 h. Afterwards, cells were centrifuged (500 g, 5min) and washed once with 200 µL FACS buffer, centrifuged and resuspended in 100-200 µL FACS buffer and assessed by flow-cytometry. The division index of eF450 labelled Teff cells was calculated using the formula log((MFI of unstimulated population)/(MFI of stimulated population))/Log(2).

**Generation of bone-marrow derived dendritic cells (BMDCs)**

The femoral and/or tibial bone(s) from double transgenic mice was/were removed under aseptic conditions. After removing adjacent tissue, bones were transferred to a 50 mL falcon tube containing 5 mL of 70 % (v/v) ethanol and immersed by shaking for 1 minute. Subsequently, ethanol was carefully decanted and femurs were washed in PBS twice. Thereafter, the distal and proximal epiphyses were removed using a scissor and the bone was longitudinally put into a PCR tube where the bottom was chopped away using scissors and put into a microcentrifuge tube with 50 µL of DC medium in place (RPMI-1640, 10 % FCS, 1x Pen/Strep/Amphotericin, L-Gln). Then, tubes were centrifuged for a few seconds (up to 3000 g) and the PCR tube and bone removed. Bone marrow cells on the bottom of the microcentrifuge tube were re-suspended and transferred into a 15 mL Falcon tube and washed once with DC medium and centrifuged (500 g, 5 minutes). Cells were then resuspended and counted after adding 2 drops of Zapoglobin II (Beckman Coulter). Cells were seeded at a concentration of 2x10^5^ cells/mL in 10 mL DC medium in 10 cm petri dishes (Sarstedt) supplemented with GM-CSF (Miltenyi) at a concentration of 20 ng/mL. Medium was changed every 2 - 3 days. Medium taken from cultures for exchange was centrifuged (500 g, 5 minutes) to avoid cell loss and the cell pellet resuspended in fresh medium supplemented with fresh GM-CSF [20 ng/mL] and returned to the suspension culture.

Cells in culture medium and cells detachable from the surface by flushing with a 10 mL pipette (loosely adherent cells) were used from day 7- 14 after culturing under these conditions for experiments.

**Sorting plus RNA isolation of FACS sorted naïve CD4^+^ T cells and differentiated T cells**

Naïve CD4^+^ T cells FACS sorted as described above were used for RNA isolation (1 x 10^6^ cells) using the RNeasy Mini Kit (Qiagen) according to the manufacturer’s recommendations. Differentiated T cells derived from FACS sorted naïve T cells cultured in 96-well round bottom plates for 5d under the conditions described above (Teff, Th-IL-2, iTreg) were collected and washed in PBS containing 0.5 % FCS and 2 mM EDTA (sorting buffer) and stained for HLA-DR, washed with sorting buffer and then resuspended in the same buffer containing 1:1000 diluted Propidium iodide (BD Biosciences). Afterwards, cells were sorted from this solution on a Sony SH800 cell sorter (Sony) for HLA-DR-/PI- cells to collect viable T lymphocytes and subsequently RNA isolated from 1 x 10^6^ of these sorted cells per condition using the RNeasy Mini Kit (Qiagen). Isolated RNA from naïve T cells and differentiated T cells was snap frozen in liquid nitrogen and then stored at -80°C until further processing by the core facility as described below. Remaining cells were stained and assessed by FACS analysis for surface and intranuclear antigens as described before with the antibodies listed in **TABLE S5.**

**Assessment of transcription factor translocation using imaging flow-cytometry**

Splenocytes from double transgenic mice [4] were isolated as described above and incubated in 96-well round bottom tissue culture plates and stimulated (2x10^5^/well) with the indicated concentrations (7.3 µM) of the Art v 1_23-36_ peptide in the presence or absence of BX-795 (Selleckchem), harmine (Sigma) or amlexanox (Selleckchem). Alternatively, cells were incubated in medium alone, PMA plus ionomycin, or with the irrelevant Bet v 1_142-153_ or Bet v 1_112-123_ peptides (7.3 µM) used as independent specificity controls in a final volume of 200 µL. Cell culture supernatants were removed after 24 and 48 h for cytokine determinations and stored at -20°C. The remaining supernatant was discarded. Cells were then stained for viability using the eFluor780 viability dye (eBioscience), incubated with anti mouse CD16/32 (Biolegend) to block free Fc-receptors, CD3 (BV421, Biolegend), CD4 (Alexa Fluor 594, Biolegend), fixed, permeabilized (eBioscience, Foxp3 TF staining buffer set) and stained for NFATc1 (AX-488) and nuclei (DRAQ5). Cells were then washed once with 100 µL Perm/Wash buffer, 200 µL Perm/Wash buffer and 200 µL DPBS and finally resuspended in 30 µL DPBS and analyzed on an ImageStreamX Mark II apparatus.

**Simultaneous assessment of transcription factors/cytokines and surface markers**

Splenocytes from double transgenic allergy mice were stimulated with the cognate Art v 1_23-36_ peptide at a concentration of 7.29 µM for 72 h in the presence of solvent (DMSO), BX-795 (1.2 µM and 0.24 µM) and under iTreg polarizing conditions (a-TGF-β [20 ng/mL], a-IL-4 [5 µg/mL], a-IFN-γ 5 µg/mL) in 96 well u-bottom plates in a final volume of 200 µL. After 72 h, 100 µL of supernatant were taken and replaced by 100 µL fresh medium to deliver a final concentration of PMA [50 ng/mL] and Ionomycin [1 µg/mL] and Monensin/Brefeldin A [1x]. Cells were stimulated for 4-6 hours and afterwards stained for viability, surface markers (**TABLE S5**) and fixed using a 50:50 mix of two different fixation buffers (IC fixation buffer, Biolegend and the eBioscience Fixation/Permeabilization buffer from the Foxp3 transcription factor buffer set, eBioscience) for 50 min. Afterwards, cells were washed twice using the eBioscience Perm/Wash buffer from the eBioscience Foxp3 transcription factor buffer set (eBioscience) and stainined using 100 µL Perm/Wash buffer containing the diluted intracellular/intranuclear antibodies (**TABLE S5**) for 50 min. Afterwards, cells were washed twice using Perm/Wash buffer and finally resuspended in 50-100 µL FACS buffer and acquired on a BD LSR Fortessa.

**Statistical analyses**

Normally distributed data were compared using parametric tests (Student's t-test or one-way ANOVA) followed by correction of alpha (Dunnett, Tukey or Holms-Sidak) using GraphPad 9.0.1 (GraphPad Software Inc., La Jolla, CA). Otherwise, the Mann-Whitney U-test or the Kruskal-Wallis test was performed, followed by Dunn’s multiple comparison testing. ns, not significant; *, p < 0.05; **, p < 0.01; ***, p <0.001.

**Data exclusions**

Grubb’s outlier test was run in prism 9.0.1 to detect and exclude significant outliers with p < 0.001.

**RNA isolation for RNA-Seq and qPCR confirmation of selected genes**

RNA was prepared from sorted cells using the Qiagen RNAeasy mini kit according to the manufacturers recommendations.

**qPCR of RNA isolated for RNA-Seq**

RNA isolated as described above was reverse transcribed using the LunaScript RT SuperMix kit (Cat. No.: E3010S, New England Biolabs) according to manufacturer’s recommendations. Quantititave real time PCR was performed using the Luna Universal qPCR Master Mix (Cat. No.: M3003L, New England Biolabs) according to the manufacturer’s recommendations.

**RNA-Seq and data analysis**

Sequencing libraries were prepared at the Core Facility Genomics, Medical University of Vienna using the NEBNext Poly(A) mRNA Magnetic Isolation Module and the NEBNext UltraTM II Directional RNA Library Prep Kit for Illumina according to manufacturer's protocols (New England Biolabs). Libraries were QC-checked on a Bioanalyzer 2100 (Agilent) using a High Sensitivity DNA Kit for correct insert size and quantified using Qubit dsDNA HS Assay (Invitrogen). Pooled libraries were sequenced on a NextSeq500 instrument (Illumina) in 1x75bp single-end sequencing mode.

Approximately 32 million reads were generated per sample.

Reads in fastq format were aligned to the mouse reference genome version GRCm38 [6] with Gencode mV23 annotations [7] using STAR aligner [8] version 2.6.1a in 2-pass mode. Reads per gene were counted by STAR, and differential gene expression was calculated using DESeq2 [9] version 1.22.2.

**SUPPLEMENTAL TableS and FIGURE LEGENDS**

**SUPPLEMENTAL Tables**

**TABLE S1. Small molecule inhibitors used in this study.**

Name, primary target, concentration, solvent, source/vendor

**TABLE S2. List of RNA-Seq by expectation maximization (RSEM) patterns.**

Shows the coding of each RSEM pattern with regards to genes similarly expressed between different groups

**TABLE S3. Gene ontology (GO) terms.**

List of GO-terms enriched for genes differentially expressed between Th-IL-2 and iTreg, using clusterProfiler.

**TABLE S4. Primers used for RTqPCR.**

Forward and reverse primer sequences for respective targets

**TABLE S5. Antibodies used in this study.**

Clone, primary target, source/vendor, application

**TABLE S6. Cytokines used in this study.**

Name, Source, Vendor

**SUPPLEMENTAL FIGURE S1.** BX795 inhibits sCD25 generation but does not impact on viability at IL-2 enhancing concentrations.

SUPPLEMENTAL FIGURE S2. BX-795 differentially modulates TCR early proximal and distal signaling events and Ca^2+^-flux.

**SUPPLEMENTAL FIGURE S3.** BX-795 stimulates IL-2 production but inhibits effector cytokine secretion by murine splenocytes.

**SUPPLEMENTAL FIGURE S4.** Neither enhanced NFATc1 translocation nor expression are mandatory for increased IL-2 levels.

**SUPPLEMENTAL FIGURE S5.** BX-795 inhibits T cell proliferation.

**SUPPLEMENTAL FIGURE S6.** BX-795 inhibits allergen-specific cytokine recall responses.

**SUPPLEMENTAL FIGURE S7.** Confirmation of RNA-Seq results for selected genes by RT-qPCR.

**SUPPLEMENTAL FIGURE S8.** BX-795 induces a CD39^+^CD62L^+^ population enriched in IL-2 and Helios but with reduced effector cytokine expression.

**SUPPLEMENTAL FIGURE S9.** BX-795 differentiated cells show mild suppressive capacities compared to iTregs.

**SUPPLEMENTAL FIGURE S10.** IL-2 is not directly required for the Th2 inhibitory effect of BX-795.

**SUPPLEMENTAL FIGURE S11.** Effects of BX-795 treatment on absolute T cell numbers in double transgenic allergy mice.

**SUPPLEMENTAL FIGURE S12.** BX-795 inhibits Foxp3^+^ iTreg polarization.

**SUPPLEMENTAL FIGURE S13.** BX-795 induces the transcription factor Helios and changes the Helios/Foxp3 balance during iTreg differentiation.

SUPPLEMENTAL FIGURE S14. BX-795 does not impact on myeloid cells other than eosinophils in murine lungs with or without mugwort pollen extract challenge.

**SUPPLEMENTAL FIGURE S15.** Gating strategy for FACS analysis of murine lung cell populations.

**SUPPLEMENTAL FIGURE S16.** BX-795 ameliorates Th2 inflammation in a murine model of HDM-induced airway hyperreactivity.

**SUPPLEMENTAL FIGURE S17.** BX-795 differentially regulates c-Maf and Fli-1.

**SUPPLEMENTAL REFERENCES**

[1] A. Cossarizza, H.D. Chang, A. Radbruch, A. Acs, D. Adam, S. Adam-Klages, W.W. Agace, N. Aghaeepour, M. Akdis, M. Allez, L.N. Almeida, G. Alvisi, G. Anderson, I. Andra, F. Annunziato, A. Anselmo, P. Bacher, C.T. Baldari, S. Bari, V. Barnaba, J. Barros-Martins, L. Battistini, W. Bauer, S. Baumgart, N. Baumgarth, D. Baumjohann, B. Baying, M. Bebawy, B. Becher, W. Beisker, V. Benes, R. Beyaert, A. Blanco, D.A. Boardman, C. Bogdan, J.G. Borger, G. Borsellino, P.E. Boulais, J.A. Bradford, D. Brenner, R.R. Brinkman, A.E.S. Brooks, D.H. Busch, M. Buscher, T.P. Bushnell, F. Calzetti, G. Cameron, I. Cammarata, X. Cao, S.L. Cardell, S. Casola, M.A. Cassatella, A. Cavani, A. Celada, L. Chatenoud, P.K. Chattopadhyay, S. Chow, E. Christakou, L. Cicin-Sain, M. Clerici, F.S. Colombo, L. Cook, A. Cooke, A.M. Cooper, A.J. Corbett, A. Cosma, L. Cosmi, P.G. Coulie, A. Cumano, L. Cvetkovic, V.D. Dang, C. Dang-Heine, M.S. Davey, D. Davies, S. De Biasi, G. Del Zotto, G.V. Dela Cruz, M. Delacher, S. Della Bella, P. Dellabona, G. Deniz, M. Dessing, J.P. Di Santo, A. Diefenbach, F. Dieli, A. Dolf, T. Dorner, R.J. Dress, D. Dudziak, M. Dustin, C.A. Dutertre, F. Ebner, S.B.G. Eckle, M. Edinger, P. Eede, G.R.A. Ehrhardt, M. Eich, P. Engel, B. Engelhardt, A. Erdei, et al., Guidelines for the use of flow cytometry and cell sorting in immunological studies (second edition). Eur J Immunol 49 (2019) 1457-1973.

[2] M. Banda, A. Bommineni, R.A. Thomas, L.S. Luckinbill, and J.D. Tucker, Evaluation and validation of housekeeping genes in response to ionizing radiation and chemical exposure for normalizing RNA expression in real-time PCR. Mutat Res 649 (2008) 126-34.

[3] M.A. Goldsmith, P.F. Dazin, and A. Weiss, At least two non-antigen-binding molecules are required for signal transduction by the T-cell antigen receptor. Proc Nat Acad of Sci USA 85 (1988) 8613-7.

[4] A. Neunkirchner, B. Kratzer, C. Kohler, U. Smole, L.F. Mager, K.G. Schmetterer, D. Trapin, V. Leb-Reichl, E. Rosloniec, R. Naumann, L. Kenner, B. Jahn-Schmid, B. Bohle, R. Valenta, and W.F. Pickl, Genetic restriction of antigen-presentation dictates allergic sensitization and disease in humanized mice. EBioMedicine 31 (2018) 66-78.

[5] M. Mahler Convenor, M. Berard, R. Feinstein, A. Gallagher, B. Illgen-Wilcke, K. Pritchett-Corning, and M. Raspa, FELASA recommendations for the health monitoring of mouse, rat, hamster, guinea pig and rabbit colonies in breeding and experimental units. Lab Anim 48 (2014) 178-192.

[6] Ensembl, mm10 murine reference genome downloaded from Ensembl (2018-09-28). <ftp://ftp.ensembl.org/pub/release-93/fasta/mus_musculus/dna/Mus_musculus.GRCm38.dna_sm.primary_assembly.fa.gz>.

[7] Gencode, Murine genome annotations downloaded from Gencode (2019-10-14). <ftp://ftp.ebi.ac.uk/pub/databases/gencode/Gencode_mouse/release_M23/gencode.vM23.chr_patch_hapl_scaff.annotation.gtf.gz>.

[8] A. Dobin, C.A. Davis, F. Schlesinger, J. Drenkow, C. Zaleski, S. Jha, P. Batut, M. Chaisson, and T.R. Gingeras, STAR: ultrafast universal RNA-seq aligner. Bioinformatics 29 (2013) 15-21.

[9] M.I. Love, W. Huber, and S. Anders, Moderated estimation of fold change and dispersion for RNA-seq data with DESeq2. Genome Biol 15 (2014) 550.

**SUPPLEMENTAL TABLES**

**TABLE S1.** Targeted drugs used in this study

| **Target** | **TCR signaling relevant target(s)** | **Drug name** | **Source** | **Concentration range used** |
| --- | --- | --- | --- | --- |
| FK506 | NFAT, Calcineurin | Tacrolimus | Selleckchem | 0.1-3 nM |
| Cyclophilin | NFAT, Calcineurin | Cyclosporin A | Novartis | 0.02-0.6 µM |
| ERK2 | ERK2 | Vx-11e | ChemieTek | 0.05 – 1.4 µM |
| PDK1/TBK1/IKKε | PDK1/TBK1/IKKε | BX-795 | Selleckchem | 0.24 – 30 µM |
| Lck | Lck | Saracatinib | Selleckchem | 0.5 – 12.5 µM |
| JNK | JNK | SP600125 | Selleckchem | 3.6 – 91 µM |
| mTORC1/mTORC2 | mTORC1/mTORC2 | AZD8055 | Selleckchem | 0.04 – 1 µM |
| mTORC1 | mTORC1 | Rapamycin | Sigma-Aldrich | 0.4 – 10 µM |
| TBK1/IKKε | TBK1/IKKε | Amlexanox | Selleckchem | 1 – 200 µM |
| DYRK | DYRK/NFAT | Harmine | Sigma-Aldrich | 10 – 20 µM |

**TABLE S2.** List of RNA-Seq by expectation maximization (RSEM) patterns:

| **Pattern #** | **Naive T cells** | **Teff** | **Th-IL-2** | **iTreg** | **# of genes with this pattern** |
| --- | --- | --- | --- | --- | --- |
| Pattern1 | 1 | 1 | 1 | 1 | NA |
| Pattern2 | 1 | 1 | 1 | 2 | 163 |
| Pattern3 | 1 | 1 | 2 | 1 | 89 |
| Pattern4 | 1 | 1 | 2 | 2 | 764 |
| Pattern5 | 1 | 2 | 1 | 1 | 1183 |
| Pattern6 | 1 | 2 | 1 | 2 | 252 |
| Pattern7 | 1 | 2 | 2 | 1 | 113 |
| Pattern8 | 1 | 2 | 2 | 2 | 4301 |
| Pattern9 | 1 | 1 | 2 | 3 | 35 |
| Pattern10 | 1 | 2 | 1 | 3 | 88 |
| Pattern11 | 1 | 2 | 2 | 3 | 155 |
| Pattern12 | 1 | 2 | 3 | 1 | 66 |
| Pattern13 | 1 | 2 | 3 | 2 | 364 |
| Pattern14 | 1 | 2 | 3 | 3 | 2052 |
| Pattern15 | 1 | 2 | 3 | 4 | 220 |

A total of 9845 protein coding genes were assigned to different patterns of differential gene expression between the four different experimental conditions (Naïve T cells, Teff, Th-IL-2, iTreg) using the rsem-run-ebseq function of the RSEM toolkit. Each gene can be part of one pattern only. Same numbers for different experimental conditions indicate similar expression of the genes in the respective pattern.

**TABLE S3.** Gene ontology (GO) terms

|  | Description^[[1]](#footnote-1)^ | GeneRatio^[[2]](#footnote-2)^ | BgRatio | pvalue | p.adjust | qvalue |
| --- | --- | --- | --- | --- | --- | --- |
| GO:0006955 | immune response | 44/282 | 429/6417 | 6,99507E-08 | 0,000172496 | 0,00014908 |
| GO:0035458 | cellular response to interferon-beta | 9/282 | 21/6417 | 9,94216E-08 | 0,000172496 | 0,00014908 |
| GO:0035456 | response to interferon-beta | 9/282 | 25/6417 | 5,91346E-07 | 0,000523262 | 0,000452229 |
| GO:0098609 | cell-cell adhesion | 28/282 | 228/6417 | 6,03184E-07 | 0,000523262 | 0,000452229 |
| GO:0019932 | second-messenger-mediated signaling | 20/282 | 144/6417 | 4,01726E-06 | 0,00278798 | 0,002409512 |
| GO:0008284 | positive regulation of cell proliferation | 32/282 | 317/6417 | 7,22877E-06 | 0,004180639 | 0,003613117 |
| GO:0071345 | cellular response to cytokine stimulus | 28/282 | 263/6417 | 1,03427E-05 | 0,005127025 | 0,004431031 |
| GO:0034097 | response to cytokine | 30/282 | 298/6417 | 1,50431E-05 | 0,005558328 | 0,004803785 |
| GO:0022610 | biological adhesion | 36/282 | 394/6417 | 1,80052E-05 | 0,005558328 | 0,004803785 |
| GO:0001775 | cell activation | 31/282 | 316/6417 | 1,80839E-05 | 0,005558328 | 0,004803785 |
| GO:0019722 | calcium-mediated signaling | 12/282 | 65/6417 | 2,04589E-05 | 0,005558328 | 0,004803785 |
| GO:0007159 | leukocyte cell-cell adhesion | 17/282 | 122/6417 | 2,06824E-05 | 0,005558328 | 0,004803785 |
| GO:0030155 | regulation of cell adhesion | 24/282 | 215/6417 | 2,08237E-05 | 0,005558328 | 0,004803785 |
| GO:0034341 | response to interferon-gamma | 10/282 | 46/6417 | 2,28783E-05 | 0,005670538 | 0,004900762 |
| GO:0071346 | cellular response to interferon-gamma | 9/282 | 39/6417 | 3,5614E-05 | 0,008238709 | 0,007120304 |
| GO:0050776 | regulation of immune response | 24/282 | 224/6417 | 4,11073E-05 | 0,008915156 | 0,007704923 |
| GO:0045321 | leukocyte activation | 28/282 | 286/6417 | 4,88741E-05 | 0,00997607 | 0,008621818 |
| GO:0006952 | defense response | 39/282 | 464/6417 | 5,33292E-05 | 0,010280682 | 0,008885079 |
| GO:0045785 | positive regulation of cell adhesion | 16/282 | 120/6417 | 6,28598E-05 | 0,011480179 | 0,009921744 |
| GO:0007155 | cell adhesion | 34/282 | 391/6417 | 8,61417E-05 | 0,014270615 | 0,012333378 |
| GO:0002682 | regulation of immune system process | 36/282 | 424/6417 | 8,6364E-05 | 0,014270615 | 0,012333378 |
| GO:0051094 | positive regulation of developmental process | 38/282 | 459/6417 | 9,30348E-05 | 0,014674119 | 0,012682107 |
| GO:0035988 | chondrocyte proliferation | 4/282 | 7/6417 | 0,000115065 | 0,017359774 | 0,015003184 |
| GO:0016477 | cell migration | 35/282 | 420/6417 | 0,00015842 | 0,022904879 | 0,01979554 |
| GO:0003197 | endocardial cushion development | 6/282 | 21/6417 | 0,000211859 | 0,029406066 | 0,025414191 |
| GO:0045597 | positive regulation of cell differentiation | 28/282 | 312/6417 | 0,000222236 | 0,029660025 | 0,025633676 |
| GO:0009607 | response to biotic stimulus | 29/282 | 329/6417 | 0,000233207 | 0,029971464 | 0,025902836 |
| GO:1903037 | regulation of leukocyte cell-cell adhesion | 14/282 | 108/6417 | 0,000244559 | 0,030307838 | 0,026193548 |
| GO:1903522 | regulation of blood circulation | 11/282 | 72/6417 | 0,000268044 | 0,030414439 | 0,026285677 |
| GO:0048870 | cell motility | 37/282 | 466/6417 | 0,000271714 | 0,030414439 | 0,026285677 |
| GO:0051674 | localization of cell | 37/282 | 466/6417 | 0,000271714 | 0,030414439 | 0,026285677 |
| GO:0022407 | regulation of cell-cell adhesion | 16/282 | 137/6417 | 0,000305116 | 0,033086068 | 0,028594633 |
| GO:0051707 | response to other organism | 28/282 | 319/6417 | 0,0003216 | 0,03366742 | 0,029097066 |
| GO:0043207 | response to external biotic stimulus | 28/282 | 320/6417 | 0,000338621 | 0,03366742 | 0,029097066 |
| GO:0002684 | positive regulation of immune system process | 26/282 | 288/6417 | 0,000341619 | 0,03366742 | 0,029097066 |
| GO:0007204 | positive regulation of cytosolic calcium ion concentration | 15/282 | 125/6417 | 0,000349287 | 0,03366742 | 0,029097066 |
| GO:0072507 | divalent inorganic cation homeostasis | 19/282 | 183/6417 | 0,000395684 | 0,037108703 | 0,032071195 |
| GO:0050679 | positive regulation of epithelial cell proliferation | 10/282 | 64/6417 | 0,000421895 | 0,038525639 | 0,033295782 |
| GO:0006935 | chemotaxis | 19/282 | 185/6417 | 0,000454091 | 0,038744821 | 0,03348521 |
| GO:0009611 | response to wounding | 17/282 | 156/6417 | 0,000454966 | 0,038744821 | 0,03348521 |
| GO:0031347 | regulation of defense response | 20/282 | 200/6417 | 0,000457792 | 0,038744821 | 0,03348521 |
| GO:0061448 | connective tissue development | 12/282 | 89/6417 | 0,000469427 | 0,038783613 | 0,033518736 |
| GO:0042330 | taxis | 19/282 | 186/6417 | 0,000486005 | 0,039219442 | 0,033895402 |
| GO:0050867 | positive regulation of cell activation | 14/282 | 116/6417 | 0,000516573 | 0,040738821 | 0,035208525 |
| GO:0009719 | response to endogenous stimulus | 33/282 | 413/6417 | 0,000531498 | 0,040984367 | 0,035420737 |
| GO:0002252 | immune effector process | 22/282 | 234/6417 | 0,000566625 | 0,04274323 | 0,036940835 |
| GO:0033627 | cell adhesion mediated by integrin | 6/282 | 25/6417 | 0,000595965 | 0,043999933 | 0,038026941 |
| GO:2000108 | positive regulation of leukocyte apoptotic process | 4/282 | 10/6417 | 0,000621395 | 0,044921704 | 0,038823581 |
| GO:0008015 | blood circulation | 17/282 | 161/6417 | 0,000656597 | 0,046497803 | 0,040185725 |
| GO:0003013 | circulatory system process | 17/282 | 162/6417 | 0,000705021 | 0,048928478 | 0,042286435 |
| GO:0002443 | leukocyte mediated immunity | 14/282 | 120/6417 | 0,000729509 | 0,049430796 | 0,042720564 |
| GO:0050868 | negative regulation of T cell activation | 8/282 | 46/6417 | 0,000765013 | 0,049430796 | 0,042720564 |
| GO:0050865 | regulation of cell activation | 19/282 | 193/6417 | 0,000768989 | 0,049430796 | 0,042720564 |
| GO:0072503 | cellular divalent inorganic cation homeostasis | 18/282 | 178/6417 | 0,00076924 | 0,049430796 | 0,042720564 |

**TABLE S4.** Primers used for RT-qPCR

| **Target gene** | **Forward primer 5’-3’** | **Reverse primer 5’-3’** | **Species** | **Source** |
| --- | --- | --- | --- | --- |
| IL-2 | TCACCAGGATGCTCACATTT | GCACTTCCTCCAGAGGTTTG | Human | Sigma |
| IFN-γ | CGAGATGACTTCGAAAAGCTG | TCAGCCATCACTTGGATGAG | Human | Sigma |
| β2-Microglobulin | TAGCTGTGCTCGCGCTACT | TCTCTGCTGGATGACGTGAG | Human | Sigma |
| Ribosomal protein S14 | TGGTGTCTGCCACATCTTTGCATC | AGTCACTCGGCAGATGGTTTCCTT | Mouse | Sigma |
| IKZF2 | GGAATCCGGCTTCCGAATGG | GTGAGAGCGTCCCTTCTTCTA | Mouse | Sigma |
| IL-4 | TGAACGAGGTCACAGGAGAA | CGAGCTCACTCTCTGTGGTG | Mouse | Sigma |
| Foxp3 | CCCATCCCCAGGAGTCTTG | ACCATGACTAGGGGCACTGTA | Mouse | Sigma |

**TABLE S5.** Antibodies used in this study

| **Specificity** | | **Clone name** | | **Species** | | **Conjugate** | | **Source** |  |
| --- | --- | --- | --- | --- | --- | --- | --- | --- | --- |
| ***Flow cytometry of murine cells*** | | | | | | | | | |
| *Dendritic cell staining panel* | | | | | | | | | |
| CD11b | | M1/70 | | rat | | APC-Cy7 | | Biolegend |  |
| CD11c | | N418 | | hamster | | PE-Cy7 | | Biolegend |  |
| Ly6G | | 1A8 | | rat | | BV605 | | Biolegend |  |
| Ly6C | | HK1.4 | | rat | | BV421 | | Biolegend |  |
| MHC-II | | M5/114.15.2 | | rat | | BV650 | | Biolegend |  |
| CD103 | | 2E7 | | hamster | | PerCP-Cy 5.5 | | Biolegend |  |
| CD24 | | M1/69 | | rat | | Alexa Fluor 488 | | Biolegend |  |
| CD64 | | X54-5/7.1 | | mouse | | BV711 | | Biolegend |  |
| CD45 | | 30-F11 | | rat | | Alexa Fluor 700 | | BD |  |
| Siglec-F | | E50-2440 | | rat | | APC | | BD |  |
| *T cells native* | |  | |  | |  | |  |  |
| CD25 | | PC61 | | rat | | BV650 | | Biolegend |  |
| CD3 | | 17A2 | | rat | | FITC | | Biolegend |  |
| CD4 | | RM4-4 | | rat | | PerCP-Cy 5.5 | | Biolegend |  |
| T-bet | | 4B10 | | mouse | | BV711 | | Biolegend | |
| GATA-3 | | L50-823 | | mouse | | PE-Cy7 | | BD |  |
| Foxp3 | | FJK-16s | | rat | | PE | | eBioscience |  |
| ROR-γt | | Q31-378 | | mouse | | Alexa Fluor 647 | | BD |  |
| *T cells re-stimulated* | | | | | | | | |  |
| CD3 | | 17A2 | | rat | | FITC | | Biolegend |  |
| CD4 | | RM4-4 | | rat | | PerCP-Cy 5.5 | | Biolegend |  |
| IFN-γ | | XMG1.2 | | rat | | BV650 | | Biolegend |  |
| IL-17a | | eBio17B7 | | rat | | APC | | Invitrogen/eBioscience |  |
| IL-13 | | eBio13A | | rat | | PE-Cy7 | | Invitrogen/eBioscience |  |
| IL-13 | | eBio13A | | rat | | PE | | Invitrogen/eBioscience |  |
| IL-10 | | JES5-16E3 | | rat | | PE | | Biolegend |  |
| IL-2 | | JES6-5H4 | | rat | | BV711 | | Biolegend |  |
| *Assessment of transcription factor translocation in CD4^+^ T cells using imaging flow-cytometry* | | | | | | | | |  |
| CD3 | | 145-2C11 | | hamster | | BV421 | | Biolegend |  |
| CD4 | | GK1.5 | | rat | | Alexa Fluor 594 | | Biolegend |  |
| Life/Dead | | - | | - | | eFluor 780 | | Thermo Fisher |  |
| Nuclear dye | | - | | - | | DRAQ5 | | Biolegend |  |
| NFATc1 | | 7A6 | | mouse | | Alexa Fluor 488 | | Biolegend |  |
| *Stimulation of CD4^+^ T cells in vitro* | | | | | | | | |  |
| CD3 (Ultra-Leaf) | | 145-2C11 | | hamster | | - | | Biolegend |  |
| CD28 (Ultra-Leaf) | | 37.51 | | hamster | | - | | Biolegend |  |
| ***Flow cytometry of human cells*** | | | | | | | | | |
| *Flow cytometric analysis of hPBMCs* | | | | | | | | | |
| CD3 | | UCHT1 | | mouse | | PerCP | | Biolegend | |
| CD4 | | RPA-T4 | | mouse | | FITC | | Biolegend | |
| CD8 | | 3B5 | | mouse | | APC | | eBioscience | |
| CD25 | | BC96 | | mouse | | PerCP-Cy 5.5 | | Biolegend | |
| CD69 | | FN50 | | mouse | | BV650 | | Biolegend | |
| CD154 (CD40L) | | 24-31 | | mouse | | BV421 | | Biolegend | |
| CD49e | | NKI-SAM-1 | | mouse | | PE | | Biolegend | |
| IFN-γ | | 4S.B3 | | mouse | | BV421 | | Biolegend | |
| IL-17a | | BL168 | | mouse | | BV711 | | Biolegend | |
| IL-2 | | MQ1-17H12 | | rat | | PE | | Biolegend | |
| ***Multiplex analysis of secreted cytokines*** | | | | | | | | |  |
| *Luminex primary antibodies (mouse):* | | | | | | | | |  |
| IL-10 | | JES5-16E3 | | rat | | - | | eBioscience |  |
| IL-2 | | JES6-1A12 | | rat | | - | | eBioscience |  |
| IL-4 | | 11B11 | | rat | | - | | eBioscience |  |
| IL-5 | | TRFK5 | | rat | | - | | eBioscience |  |
| IL-13 | | eBio13A | | rat | | - | | eBioscience |  |
| IL-17 | | 17CK15A5 | | rat | | - | | eBioscience |  |
| GM-CSF | | MP1-22E9 | | rat | | - | | eBioscience |  |
| IFN-γ | | AN-18 | | rat | | - | | eBioscience |  |
| TNF-α | | 1F3F3D4 | | rat | | - | | eBioscience |  |
| Luminex secondary (biotinylated) antibodies (mouse): | | | | | | | | |  |
| IL-10 | | JES5-2A5 | | rat | | biotin | | eBioscience |  |
| IL-2 | | JES6-5H4 | | rat | | biotin | | eBioscience |  |
| IL-4 | | BVD6-24G12 | | rat | | biotin | | eBioscience |  |
| IL-5 | | TRFK4 | | rat | | biotin | | eBioscience |  |
| IL-13 | | eBio1316H | | rat | | biotin | | eBioscience |  |
| IL-17 | | eBio17B7 | | rat | | biotin | | eBioscience |  |
| GM-CSF | | MP1-31G6 | | rat | | biotin | | eBioscience |  |
| IFN-γ | | R4-6A2 | | rat | | biotin | | eBioscience |  |
| TNF-α | | XT3/XT22 | | rat | | biotin | | eBioscience |  |
| Luminex primary antibodies (human): | | | | | | | | |  |
| IL-10 | | JES3-9D7 | | rat | | - | | eBioscience |  |
| IL-2 | | MQ1-17H12 | | rat | | - | | eBioscience |  |
| IL-4 | | 8D4-8 | | rat | | - | | eBioscience |  |
| IL-5 | | TRFK5 | | rat | | - | | Biolegend |  |
| IL-13 | | JES10-5A2 | | rat | | - | | Biolegend |  |
| IL-17a | | BL23 | | mouse | | - | | Biolegend |  |
| GM-CSF | | BVD2-23B6 | | rat | | - | | Biolegend |  |
| IFN-γ | | MD-1 | | mouse | | - | | Biolegend |  |
| sCD25 | | B-B10 | | mouse | | - | | eBioscience |  |
| Luminex secondary (biotinylated) antibodies (human): | | | | | | | | |  |
| IL-10 | | JES3-12G8 | | rat | | biotin | | Biolegend |  |
| IL-2 | | polyclonal | | rabbit | | biotin | | eBioscience |  |
| IL-4 | | MP4-25D2 | | rat | | biotin | | eBioscience |  |
| IL-5 | | JES1-5A10 | | rat | | biotin | | Biolegend |  |
| IL-13 | | Poly5020 | | rabbit | | biotin | | Biolegend |  |
| IL-17a | | Poly5189 | | goat | | biotin | | Biolegend |  |
| GM-CSF | | BVD2-21C11 | | rat | | biotin | | Biolegend |  |
| IFN-γ | | 4S.B3 | | mouse | | biotin | | Biolegend |  |
| sCD25 | | M-A251 | | mouse | | biotin | | Biolegend |  |
| ***Antibodies for Western blot analysis:*** | | | | | | | | |  |
| Lck | | 3A5 | | mouse | | - | | Santa Cruz Biotechnology (sc-433) |  |
| pan ζ-chain | | 1D4 | | mouse | | - | | BD |  |
| pY142 ζ-chain | | K25-407.69 | | mouse | | - | | BD |  |
| GAPDH | | 14C10 | | rabbit | |  | | CST |  |
| pY416-Src | | D49G4 | | rabbit | |  | | CST |  |
| pT202/pY204 p44/42 MAPK (Erk1/2) | | D13.14.4E | | rabbit | | - | | CST |  |
| p44/42 MAPK (Erk1/2) | | 3A7 | | mouse | | - | | CST |  |
| pan pY | | 4G10 | | mouse | | HRP | | Merck Millipore |  |
| ***Neutralizing antibodies and isotype controls for polarization experiments:*** | | | | | | | | |  |
| TGF-β | | 1D11 | | mouse | | - | | Bioxcell |  |
| Isotype control | | MOPC-21 | | mouse | | - | | Bioxcell |  |
| IL-2 | | JES6-1A12 | | rat | | - | | Invitrogen/eBioscience |  |
| IL-2 | | S4B6-1 | | rat | | - | | Bioxcell |  |
| Isotype control | | RTK2758 | | rat | | - | | Biolegend |  |
| IFN-γ | | XMG1.2 | | rat | | - | | Bioxcell |  |
| IL-4 | | 11B11 | | rat | | - | | Bioxcell |  |
| IL-12 | | R1-5D9 | | rat | | - | | Bioxcell |  |
|  | |  | |  | |  | |  |  |
| ***Antibodies for FACS sort of naïve T cells, differentiated T cells and Helios/Foxp3/CD39/CD73 assessment*** | | | | | | | | |  |
| CD4 | | RM4-5 | | rat | | PE-Cy7 | | BD |  |
| CD4 | | GK1.5 | | rat | | BV711 | | Biolegend |  |
| CD8 | | 53-6.7 | | rat | | AF700 | | Biolegend |  |
| CD44 | | IM7 | | rat | | FITC | | Biolegend |  |
| CD62L | | MEL-14 | | rat | | PE | | Biolegend |  |
| CD25 | | PC61 | | rat | | APC/BV650 | | Biolegend |  |
| HLA-DR | | L243 | | Mouse | | FITC | | Biolegend | |
| CD3e | | 145-2C11 | | rat | | APC-eF780 | | eBioscience |  |
| CD39 | | 24DMS1 | | rat | | PerCP-eF710/  PE-Cy7 | | eBioscience |  |
| CD73 | | TY/11.8 | | rat | | BV421 | | Biolegend |  |
| Foxp3 | | FJK-16s | | rat | | APC | | eBioscience |  |
| Foxp3 | | FJK-16s | | rat | | PE | | eBioscience |  |
| Helios | | 22F6 | | Armenian hamster | | PE/Dazzle 594 | | Biolegend |  |
| ***Antibodies for simultaneous surface marker, transcription factor and cytokine staining*** | | | | | | | | |  |
| CD3e | | 145-2C11 | | rat | | APC-eF780 | | eBioscience |  |
| CD4 | | RM4-5 | | rat | | Alexa Fluor 700 | | BD |  |
| CD39 | | 24DMS1 | | rat | | PerCP-eF710 | | eBioscience |  |
| CD73 | | TY/11.8 | | rat | | BV421 | | Biolegend |  |
| CD62L | | MEL-14 | | rat | | BV786 | | Biolegend |  |
| CD39 | | 24DMS1 | | rat | | PE-Cy7 | | eBioscience |  |
| CD73 | | TY/11.8 | | rat | | BV421 | | Biolegend |  |
| T-bet | | 4B10 | | mouse | | Alexa Fluor 488 | | Biolegend |  |
| GATA-3 | | L50-823 | | mouse | | PE-Cy7 | | BD |  |
| Foxp3 | | FJK-16s | | rat | | APC | | eBioscience |  |
| Helios | | 22F6 | | Armenian hamster | | PE/Dazzle 594 | | Biolegend |  |
| IL-2 | | JES6-5H4 | | rat | | BV711 | | Biolegend |  |
| IL-13 | | eBio13A | | rat | | PE | | Invitrogen/eBioscience |  |
| IFN-γ | | XMG1.2 | | rat | | BV650 | | Biolegend |  |

**TABLE S6.** Cytokines used in this study

| **Cytokine/Growth factor** | **Host species produced** | **Source** |
| --- | --- | --- |
| Recombinant human TGF-β1 | HEK293 | Peprotech |
| Murine GM-CSF | E. coli | Miltenyi |
| Recombinant murine IL-4 | E. coli | Peprotech |
| Recombinant murine IL-12 | CHO | Peprotech |
| Recombinant murine IL-2 | E. coli | Peprotech |

**FIGURE LEGENDS ONLINE SUPPLEMENT**

**FIGURE S1.** BX-795 inhibits sCD25 generation but does not impact on viability at IL-2 enhancing concentrations. Shown are **A**, sCD25 levels in supernatants of Jurkat E6-1 T cells stimulated for 24 h in 96-well flat bottom plates coated with CD3 mAb OKT3 (colored bars) in the presence or absence of the indicated targeted drugs at the indicated final concentrations C1 – C3 (see inset table, C1 red, C2 green, C3 blue). Magenta bar shows sCD25 level in the supernatant of Jurkat E6-1 T cells solely cultured with solvent (DMSO) in CD3 coated wells. Non-stimulated and solvent only treated cells are shown as right most bar (white). **B**, Jurkat E6-1 T cells stimulated as in **A** were stained with propidium iodide (PI) and annexin-V FITC and analyzed by flow-cytometry. The percentages of PI^neg^Annexin-V^neg^ cells of the ungated population (viable cells) are shown. Data show mean values ± SEM of 4 independently performed experiments (except two for amlexanox and three for rapamycin) tested in triplicates (black bars). Magenta bars show CD3 stimulated cells in medium, white bars show cells in medium alone without stimulus (right most bar). One-way ANOVA with Dunnett’s correction for multiple comparisons against CD3 stimulated cells in solvent (mean of control indicated by dotted line). Only statistically significant changes compared to CD3 stimulated cells in medium are indicated with *, p < 0.05; **, p < 0.01; ***, p < 0.001; ****, p < 0.0001.

FIGURE S2. BX-795 differentially modulates TCR early proximal and distal signaling events and Ca^2+^-flux. Jurkat E6-1 A and primary human T cells B were Indo-labeled, rested and stimulated at 37°C with TCR/CD3 cross-linking antibodies as described in the Supplemental Methods above. Shown is the mean Indo-1 violet:blue ratio of the recorded singlet core over time, with baseline signals (first 60 seconds) followed by stimulation-induced fluxing, and concluded with addition of the ionomycin labeling control. Shown are data of one out of three (Jurkat E6-1) or one (primary T cells) representative experiments performed. Area under the curve (AUC) Ca^2+^-flux statistics were calculated as described in Supplemental Materials and Methods, and Ca^2+^-flux induction of BX-795 samples was normalized to maximal induction observed in DMSO samples. C, shown are protein tyrosine phosphorylation patterns in Jurkat E6-1 cells (1.5 x 10^6^ per sample), which were pre-incubated with DMSO (solvent) or BX-795 at the indicated concentrations for 90 minutes and subsequently incubated at 37°C with the CD3ε cross-linking MEM-92 antibody for the indicated periods of time. “0” refers to cells incubated at 37°C in the absence of stimulating mAb for 10 minutes. Cell lysates were separated by SDS-PAGE, blotted onto PVDF membranes and incubated with HRP conjugated 4G10 antibody followed by chemiluminescence-based detection of bound antibody. Subsequently, membranes were stripped and re-probed with a rabbit anti-GAPDH antibody followed by incubation with HRP-conjugated goat anti-rabbit secondary antibody and chemiluminescence detection of bound antibodies. Shown is one representative Western blot out of two independently performed experiments. Positions of molecular mass standards are indicated. kDa, kilodalton. D to F, shown are protein phosphorylation patterns in Jurkat E6-1 cells (1.5 x 10^6^), pre-incubated, stimulated and processed for immunoblotting as described above. PVDF-membranes were probed with phospho-specific antibodies as indicated and developed with HRP-conjugated secondary antibodies. Subsequently, membranes were stripped and re-probed with pan-protein specific antibodies. Shown are Western blots of three independently performed experiments. Densitometric analyses of specific signals is shown in G, with the maximum signal for each molecule normalized to stimulation in DMSO (after 1 to 3 minutes). Significance of signal reduction due to the presence of BX-795 was analyzed by Student’s t-test.

FIGURE S3. BX-795 stimulates IL-2 production but inhibits effector cytokine secretion by murine splenocytes. Shown are A, IL-2; B, IL-4; C, IL-5; D, IL-13; E, IL-10; F, GM-CSF; G, IL-17 and H, IFN-γ levels in supernatants of splenocytes (2x10^5^ cells/well) from TCR/DR1 double transgenic mice which had been incubated in 96-well round bottom tissue culture plates with Art v 1_23-36_ peptide (22 µM) in the presence (black squares) or absence (white squares) of 6 µM BX-795 for 72 hours. All cultures contained DMSO at a final concentration of 0.25 % (v/v). I, shown is a heatmap which summarizes the relative changes of secreted cytokine levels of drug treated samples as compared to non-drug treated samples (both stimulated) as determined in the supernatants of splenocytes which had been incubated with three indicated antigen (Art v 1_23-36_) concentrations for the indicated three time points. Red color indicates BX-795-dependent cytokine increase; blue color indicates BX-795-dependent cytokine decrease.

The non-stimulated baseline levels for the indicated cytokines for medium plus DMSO were (mean±SEM): IL-2: 41.8±16.1; IL-4: 1.2±0.4; IL-5: 1.5±0.6; IL-13: 8.24±4.0; IL-10, 46.4±18.8; IFN-γ: 191.8±171; IL-17A: 67.9±44.7 and GM-CSF: 8.7±3.6 pg/mL, respectively.

A to H, data show the summary (mean values ± SEM) of five (except four for IFN-γ) independently performed experiments with freshly isolated splenocytes for each experiment and I the means of the ratios of drug treated versus control treated cells. A to H, two-tailed Mann-Whitney U-test comparing drug treated with non-treated cells. Only statistically significant changes are indicated with *, p < 0.05; **, p < 0.01.

**FIGURE S4.** Neither enhanced NFATc1 translocation nor expression are mandatory for increased IL-2 levels. **A** to **C**, splenocytes (2x10^5^ cells) from double transgenic mice were stimulated in 96-well U-bottom plates in a final volume of 200 µL for 24 and 48 h with the major mugwort pollen allergen peptide Art v 1_23-36_ [7.29 µM] in the presence of the indicated drugs (amlexanox = AMLX, harmine = HARM, BX-795 = BX, numbers indicate the concentrations [µM]) all solubilized in DMSO (0.25 % v/v). Major birch pollen allergen peptide Bet v 1_142-153_ [7.29 µM] (Bet v 1) and non-stimulated cells treated with solvent only (DMSO) served as controls. After 24 and 48 h, supernatant was collected and analyzed for cytokine concentrations by multiplexing. Cells were first stained for CD3, CD4 expression and viability, then they were fixed, permeabilized and stained i.c. for NFATc1 (AX-488) and nuclei (DRAQ5). Cells were analyzed on an ImageStreamX Mark II apparatus. **A**, shown are the gating and analysis strategies for determining NFATc1 translocation by applying a similarity score for NFATc1 and DRAQ5 overlap. Representative images for selected similarity scores are shown. Shown are typical pictures of cells in the bright field channel and different fluorophore channels for the markers CD3, CD4, NFATc1 and DRAQ5 with a high similarity score between the channel for NFATc1 and the nuclear dye DRAQ5 (2.12, upper panel) and images of a cell with a low similarity score (-2.29, lower panel).

**B**, bar graphs show (mean ± SEM) the percentages of NFATc1^+^ cells at 24 h and 48 h as determined by imaging flow-cytometry (left panel), the percentages of NFATc1 translocated cells (defined as cells with a similarity score > 1 between NFATc1 and DRAQ5) of NFATc1^+^ cells (middle panel) and the corresponding levels of IL-2 in culture supernatants (right panel). Each bar graph contains a dotted line indicating the mean of the stimulated control population (DMSO, grey-shaded bar). **C**, data show results as in **B** but for DMSO and BX-795 [0.24 µM] only stimulated with Art v 1_23-36_. Results from each individual experiment are connected with lines to indicate corresponding values from each experiment. A dotted line indicates the mean of the stimulated control population (DMSO). Data show **A**, representative FACS plots and images of one out of three performed experiments (DMSO, 48 h); **B**, the mean ± SEM of pooled data from three independently performed experiments and **C**, the individual values of these 3 experiments for selected conditions. Statistically significant values are shown as *, p < 0.05; ns, non-significant; two-tailed paired Student’s t-test.

**FIGURE S5.** BX-795 inhibits T cell proliferation. **A**, human PBMCs (1x10^5^ /well) were stimulated for 72 h with SEA [50 ng/mL] or soluble OKT3 [1 µg/mL] mAb for 72 h in 96-well round bottom plates. After 72 h 100 µL of supernatant were taken and supplemented with fresh media and methyl-^3^H-thymidine. Cells were harvested 18 h after pulsing. Figure shows proliferation [cpm] as assessed by methyl-^3^H-thymidine uptake upon stimulation with soluble CD3 mAb (left figure) and SEA (right figure) at different BX-795 concentrations; “0” indicates cells treated with solvent only. **B**, splenocytes (2x10^5^/well) from double transgenic allergy mice were stimulated for the indicated time periods using the immunodominant mugwort peptide Art v 1_23-36_. Proliferation was assessed after the indicated time points as in **A**. Figure shows **A**, the proliferation of hPBMCs after 72 h in the presence of different BX-795 concentrations or solvent only and **B**, stimulated and non-treated and stimulated splenocytes after 24, 48 and 72 h at different concentrations of the stimulating peptide. Also shown is the proliferation in response to the positive control PMA/ionomycin. **C**, proliferation data from a dose finding experiment for BX-795 is shown. Splenocytes from double transgenic mice were stimulated with the immunodominant mugwort peptide Art v 1_23-36_ at a concentration of 1 µM in the presence of different BX-795 concentrations for 24 h and then pulsed with methyl-^3^H-thymidine. Also shown are the proliferation data for non-drug treated splenocytes, non-stimulated splenocytes and mock peptide (Bet v 1_112-123_) stimulated splenocytes.

Data show **A**, mean values ± SEM of two independently performed experiments with 6 different healthy human donors; **B**, mean values ± SEM from 4 independently performed experiments and **C**, representative data from two independently performed experiments mean values ± SEM. The data in **B** have been deducted for the background proliferation of non-stimulated cells. Background proliferation in **A** to **C** was usually < 5000 cpm. **A**, one way ANOVA with Dunnett’s correction for multiple comparisons, all comparisons performed to the non-drug treated and stimulated group and **B**, multiple t-tests between each stimulation condition with and without drug with no correction for multiple comparisons. Statistically significant results are indicated with *, p < 0.05; **, p < 0.01; ***, p < 0.001; ns, not significant.

**Figure S6.** BX-795 inhibits allergen-specific cytokine recall responses. MACS sorted naïve CD4^+^ T cells of double transgenic allergy mice on the C57BL/6 background were cultured under Th2 polarizing conditions in the presence of Art v 1 for 5 days (Primary stimulation), which was followed by another 3 day culture period in the presence of cytokines and blocking antibodies in the absence of Art v 1. Afterwards, cells were re-stimulated (Secondary stimulation) in 96-well round bottom plates at a concentration of 1x10^5^ cells/well together with 1x10^5^ BMDCs and 1 µg/mL of recombinant Art v 1 together with BX-795 or solvent as control at the indicated concentrations. As a stimulation control, cells were cultured with solvent and BMDCs without Art v 1 for the same time period as the secondary stimulated cells. Graphs show **A**, representative FACS plots showing the FACS-gating strategy for analyzing IL-2^-^/IL-13^+^, IL-2^+^/IL-13^+^ double positive cells, IL-2^+^/IL-13^-^ cells, total IL-2^+^ and total IL-13^+^ cells, IL-2^-^/IFN-γ^+^, IL-2^+^/IFN-γ^+^, IL-2^+^/IFN-γ^-^ and total IFN-γ^+^ cells as well as a histogram overlap of CD3^+^ cells showing proliferation dye eF450 intensity normalized to mode after secondary Art v 1 stimulation and treated with DMSO (red) or BX-795 at 1.2 µM (blue). Graphs show **B** to **F** and **H** to **K** the percentage of cytokine producing cells after short term stimulation with PMA/ionomycin at the end of the secondary stimulation in the presence of protein transport inhibitors monensin/brefeldin A for 4-6 h as determined by flow cytometry. **G**, the division index of the indicated populations calculated as Log(mean fluorescence intensity (MFI) of eF450-dye of non-divided cells/MFI of eF450-dye of CD3^+^)/Log(2) is shown. Data show mean values ± SEM of three independently performed experiments. One-way ANOVA with Dunnett’s correction for multiple comparisons, all comparisons performed against the re-stimulated and solvent treated group. Statistically significant changes are indicated with *, p < 0.05; **, p < 0.01; ns, non-significant.

**FIGURE S7.** Confirmation of RNA-Seq results for selected genes by RT-qPCR. RNA isolated from samples used for RNA-Seq shown in **FIGURE 4** and **FIGURE 5** was reverse transcribed and analysed for gene expression using RT-qPCR. Shown is the ratio of the Cq values for **A,** Foxp3; **B**, IL-4 and **C**, IKZF2 in relation to the housekeeping gene Ribosomal protein S14 (S14). The Cq values for the housekeeping gene S14 is shown in **D**. The primer sequences used are listed in **TABLE S4**. Shown are mean values from 3-4 independently performed experiments ± SEM. One-way ANOVA with Dunnett’s correction for multiple comparisons comparing each group against Th-IL-2. Statistically significant changes are indicated with *, p < 0.05; **, ns, non-significant.

**Figure S8.** BX-795 induces a CD39^+^CD62L^+^ population enriched in IL-2 and Helios but with reduced effector cytokine expression. **A** to **G,** splenocytes from double transgenic mice were stimulated with the cognate peptide Art v 1_23-36_ [7.3 µM] for 72 h in the presence of solvent (DMSO), BX-795 at the indicated concentrations (BX 1.2 µM and BX 0.24 µM respectively) and under iTreg polarizing conditions (TGF-β [20 ng/mL], a-IL-4 [5 µg/mL] and a-IFN-γ [5 µg/mL]) in 96 well u-bottom plates in a final volume of 200 µL. After 72 h, 100 µL of supernatant were taken and replaced by 100 µL fresh medium to deliver a final concentration of PMA [50 ng/mL] and Ionomycin [1 µg/mL] and Monensin/Brefeldin A [1x]. Cells were stimulated for 4-6 hours and afterwards stained for viability, surface markers, fixed and stained for intracellular/intranuclear markers using a 50:50 mix of two different fixation buffers (Biolegend IC fixation and eBioscience Fixation /Permeabilization buffer from the Foxp3 transcription factor buffer set). **A** to **F**, bar graphs show the percentage of **A**, CD62L^+^ cells among CD3^+^CD4^+^ cells; **B**, the percentage of CD39^+^CD62L^+^ cells among CD3^+^CD4^+^ cells; **C**, percentage of CD3^+^CD4^+^CD39^+^CD62L^+^IL-2^+^ among CD3^+^CD4^+^ cells; **D**, percentage of CD3^+^CD4^+^CD39^+^CD62L^+^Helios^+^ cells among CD3^+^CD4^+^ cells; **E**, the percentage of IL-13^+^ positive cells among CD3^+^CD4^+^CD39^+^CD62L^+^ cells and **F**, the percentage of IFN-g^+^ cells among CD3^+^CD4^+^CD39^+^CD62L^+^ cells.

**G**, representative FACS contour plots are shown from one representative experiment for DMSO treated cells and cells treated at a BX-795 concentration of 1.2 µM (BX 1.2 µM). Black contour plots show gating of viable CD3^+^CD4^+^ cells for individual markers in dual parameter plots CD39/CD73/CD62L vs IL-2. Blue population shows CD3^+^CD4^+^CD39^+^CD62L^+^ positive cells derived from boolean operations on the gates individual gates shown in black contour plots. An overlay between viable CD3^+^CD4^+^ (red) and the CD3^+^CD4^+^CD39^+^CD62L^+^ population is shown in a dual parameter dot plot (CD39 vs CD62L). Expression of marker genes (IL-2, Helios, IL-13 and IFN-g) of the CD3^+^CD4^+^CD39^+^CD62L^+^ population is shown. Numbers in quadrants indicate percent of the parent population. Data show **A** to **F**, representative bar graphs with means from each experiment shown as dot and overall mean ± SEM of three independently perfomed experiments with triplicates for each experiment. One-way ANOVA comparing all conditions against DMSO with Dunnett’s correction for multiple comparisons. Only significant results are indicated with *, p<0.05, **; p<0.01; ***, p<0.001. **G**, representative FACS plots from one out of three independently performed experiments are shown.

**FIGURE S9.** BX-795 differentiated cells show moderate suppressive capacities compared to iTregs. **A** to **B**, FACS sorted naïve CD4^+^ (1 x 10^5^/well) T cells from double transgenic mice were stimulated with plate-bound CD3 [coated with 100 µL 1 µg/mL] and soluble CD28 [3 µg/mL] in the presence of BX-795 [0.24 µM and 0.48 µM] (Th-IL-2 0.24 and Th-IL-2 0.48) or under iTreg polarizing conditions (TGF-β, anti-IL-4, anti-IFN-γ) (iTreg). After 120 h, cells were collected and resuspended in sorting buffer (PBS, 2mM EDTA, 0.5 % FCS) containing 1:1000 diluted PI. Cells were sorted for FSC/SSC characteristics and PI negativity. These cells were labelled with the proliferation dye eF670. CD4^+^ T cells from splenocytes of double transgenic mice were enriched using the CD4^+^ T cell enrichment kit and afterwards FACS sorted for FSC/SSC characteristics and CD4^+^CD25^-^ and labelled with the proliferation dye eF670 or eF450 (Teff). T cells labelled with the proliferation dye eF670 were incubated with co-activated 5 x 10^4^ of the FACS-sorted CD4^+^CD25^-^ effector T cells labelled with the eF450 dye in the ratios indicated in **B** and stimulated in a final volume of 200 µL with CD3/CD28 Mouse T activator beads (8 x 10^5^ beads per well) or left non-stimulated without beads in 96-well round bottom plates for 96 h. Afterwards, cells were assessed by flow-cytometry. In **A**, representative histograms are shown for the indicated ratio (upper panel) and representative FACS plots of the indicated cell populations co-activated with FACS sorted effector T cells (lower panel) at a ratio of 1:2 and 1:1 (non-stimulated). In **B**, the division index of eF450 labelled Teff cells was calculated using the formula log((MFI of unstimulated population)/(MFI of stimulated population))/Log(2). Data show results from one out of two independently performed experiments **A** and the mean ± SEM of two independently performed experiments with duplicates for each experiment **B**.

**FIGURE S10.** IL-2 is not directly required for the Th2 inhibitory effect of BX-795. **A** to **D**, bar graphs show the concentrations of **A**, IL-2; **B**, IL-4; **C**, IL-5 and **D**, IL-13 as determined by multiplex-analysis. Briefly, naïve CD4^+^ T cells [1x10^5^/well] were co-cultured with BMDCs [2x10^4^/well] and recombinant Art v 1 protein [1 µg/mL] in the presence or absence of BX-795 [0.24 µM] or iTreg polarizing conditions (TGF-β, anti-IL-4, anti-IFN-γ). Blocking-antibodies against IL-2 (JES6-1A12, S4-B6) and/or TGF-β (TGF-b) or the respective isotype controls were added at the beginning of the 120 h incubation period at a final concentration of 10 µg/mL in all permutations as indicated. After 120 hours, supernatants were harvested and cytokines analyzed by multiplexing. Data show **A** to **D,** mean values ± SEM of two to three independently performed experiments performed in duplicates. **A** to **D**, one-way ANOVA comparing each Th-IL-2 condition containing blocking antibodies with the Th-IL-2 isotype condition. The p-value is indicated by ***, p < 0.001, only significant results are shown.

FIGURE S11. Effects of BX-795 treatment on absolute T cell numbers in double transgenic allergy mice. Double transgenic allergy mice were treated as described in Figure 5 and lung cell populations were analyzed by flow-cytometry. Shown are the absolute amounts of A, GATA-3^+^, IL-4^+^ and IL-13^+^; B, T-bet^+^ and IFN-γ^+^; C ROR-γt^+^ and IL17^+^; D, Foxp3^+^, IL-2^+^ and IL-10^+^ CD4^+^ T cells per lung in million cells (mio) as determined by FACS analyses of lung homogenates. Data show the mean ± SEM of pooled results from two independently performed experiments containing 8-9 mice per group in total. The indicated p-values were calculated using one-way ANOVA with Dunnett’s correction for multiple comparisons comparing against MPE challenged and vehicle control (DMSO) treated. Only statistically significant changes are indicated with *, p < 0.05; **, p < 0.01.

**FIGURE S12.** BX-795 inhibits Foxp3^+^ iTreg polarization. **A** to **D,** FACS sorted naïve CD4^+^ T cells (1 x 10^5^/well) from double transgenic mice were incubated with BMDCs (2 x 10^4^/well), rArt v 1 protein (1 µg/mL), hrTGF-β [20 ng/mL], α-IL4 [5 µg/mL] and α-IFN-γ [5 µg/mL] in the presence of BX-795 (0.24 µM) or solvent (DMSO) for 5 days. Shown are the percentages of **A**, CD73^+^ CD4^+^ ; **B**, CD4^+^Foxp3^+^, **C** CD4^+^CD25^+^Foxp3^+^ of T cells as determined by FACS analyses. **D**, representative contour plots from cells treated as in **A** to **C** are depicted for drug treated and solvent treated cells.

Data in **A** to **C** show mean values ± SEM from triplicates of two (BX-795 0.048 and 1.2 µM) and three (BX-795 0 and 0.24 µM) independently performed experiments. One-way ANVOA with Dunnett’s correction for multiple comparisons, all comparisons were performed against the vehicle (DMSO) only treated group (BX-795 = 0 µM). *, p < 0.05; **, p < 0.01; ns = p > 0.05.

**FIGURE S13.** BX-795 induces the transcription factor Helios and changes the Helios/Foxp3 balance during iTreg differentiation. **A** to **B**, FACS sorted naïve CD4^+^ T cells (1 x 10^5^/well) from double transgenic mice were incubated with BMDCs (2 x 10^4^/well), rArt v 1 (Art v 1) protein (1 µg/mL) or no antigen in the presence of BX-795 (0.24 µM) or solvent only (DMSO) with or without hrTGF-β [20 ng/mL], α-IL4 [5 µg/mL] and α-IFN-γ [5 µg/mL], for 120 h. **A** to **B**, shown are the percentages of Helios^+^CD3^+^CD4^+^ and Foxp3^+^CD3^+^CD4^+^ cells as determined by FACS analysis of naïve CD4^+^ T cells at day 0 (naïve T cells 0h) and of cells after 120 h (Teff, Th-IL-2, iTreg, iTreg plus BX). **C**, representative contour plots from cells treated as described in **A** to **B**. Data in **A** to **B** show mean values ± SEM of three to six independently performed experiments, One-way ANOVA with Tukey’s multiple comparisons test comparing each group against each other. Statistically significant differences are indicated with *, p < 0.05; **, p < 0.01; ***, p < 0.01, ns, not significant.

FIGURE S14. BX-795 does not impact on myeloid cells other than eosinophils in murine lungs with or without mugwort pollen extract challenge. Double transgenic allergy mice were treated as described in Figure 5 and lung cell populations were analyzed by flow-cytometry. Shown is the relative amount of A, alveolar macrophages (alveolar Macs); B, neutrophils (Neutros); C, eosinophls (Eos); D, CD11b^+^ dendritic cells (CD11b+); E, intersititial Macrophages (Interstitial Macs); F, Ly6C^+^ monocytes/macrophages (Ly6C+ monos_macs) and G, Ly6C^-^ monocytes/macrophages (Ly6C- monos_macs) of viable cells in % as determined by FACS analysis of lung homogenate. Data show the mean ± SEM of pooled data from two independently performed experiments containing 8-10 mice per group. The indicated p-values were calculated using one-way ANOVA with Dunnett’s correction for multiple comparisons comparing against MPE challenged and vehicle control (DMSO) treated. Only statistically significant changes are indicated with *, p < 0.05.

**FIGURE S15.** Gating strategy for FACS analysis of murine lung cell populations. Shown is the gating strategy for the FACS analysis of different lung cell populations from *in vivo* experiments using mice sensitized and challenged with extract or PBS together with solvent or BX-795.

**FIGURE S16.** BX-795 ameliorates Th2 inflammation in a murine model of HDM-induced airway hyperreactivity. **A**, Shown is the treatment protocol indicating the time points and dosage of house-dust-mite extract (HDM) and BX-795 exposure of wildtype C57BL/6 mice. Shown are the percentages of **B**, Eosinophils; **C**, GATA-3^+^ and IL-13^+^ T cells; **D**, ROR-γt^+^ and IL-17^+^ T cells; **E**, Foxp3^+^ and IL-2^+^ and **F**, IFN-γ^+^ T cells in the lung of mice sensitized by i.t. administration of HDM or placebo (PBS) in the presence or absence of locally applied BX-795 as determined by FACS analyses. **G**, shown are representative lung sections stained for periodic acid-Schiff (PAS, left panel) or hematoxylin eosin (HE, right panel) of mice of the indicated treatment groups. **B** to **F,** data shows the mean ± SEM of pooled data from two (**C** to **E**, % GATA-3^+^/ROR-γt^-^, % GATA-3^−^/ROR-γt^+^, % IL-2^+^ and % Foxp3^+^) containing *n = 9-12* both PBS groups, *n = 11-13* HDM plus BX-795 and *n = 10-12* HDM plus DMSO mice and three independently performed experiments **B**; **C** to **F**, % IL-13^+^, % IL-17^+^, % IFN-γ^+^ containing *n = 15* PBS plus DMSO, *n = 14* PBS plus BX-795, *n = 18* HDM plus BX-795 and *n = 16-17* HDM plus DMSO mice all comprising male and female mice. The indicated p-values were calculated using one-way ANOVA with Dunnett’s correction for multiple comparisons comparing against HDM challenged and vehicle control (DMSO) treated. Only statistically significant changes are indicated with *, p < 0.05; **, p < 0.01; ***, p<0.001.

**FIGURE S17.** BX-795 differentially regulates c-Maf and Fli-1.

Shown are bar graphs summarizing the expression of the genes Maf and Fli-1 for the four experimental conditions (naïve T cells, Teff, Th-IL-2 and iTreg) as transcripts per million (TPM). Data show **A** and **B**, the mean value ± SEM of four independently performed experiments. One-way ANOVA with Dunnett’s correction of multiple comparisons. All comparisons performed against the condition Th-IL-2. Statistically significant changes are indicated with ****, p < 0.001. ns, non-significant.

**FIGURE S1**

**FIGURE S2**

**FIGURE S3**

**FIGURE S4**

**FIGURE S5**

**FIGURE S6**

**FIGURE S7**

**FIGURE S8**

**FIGURE S9**

**FIGURE S10**

**FIGURE S11**

**FIGURE S12**

**FIGURE S13**

**FIGURE S14**

**FIGURE S15**

**FIGURE S16**

**FIGURE S17**

1. Name of the Gene ontology (GO) term containing a defined set of genes according to clusterProfiler [↑](#footnote-ref-1)
2. Ratio between the number of differentially expressed genes and genes contained in the respective GO term [↑](#footnote-ref-2)
